# Ecomorphological characterization of murines and non-arvicoline cricetids (Rodentia) from south-western Europe since the latest Middle Miocene to the Mio-Pliocene boundary (MN 7/8–MN13)

**DOI:** 10.7717/peerj.3646

**Published:** 2017-09-25

**Authors:** Ana R. Gomez Cano, Yuri Kimura, Fernando Blanco, Iris Menéndez, María A. Álvarez-Sierra, Manuel Hernández Fernández

**Affiliations:** 1 Institut Català de Paleontologia Miquel Crusafont, Universitat Autónoma de Barcelona, Cerdanyola del Vallès, Barcelona, Spain; 2 Transmitting Science, Barcelona, Spain; 3 Department of Geology and Paleontology, National Museum of Nature and Science, Tokyo, Japan; 4 Departamento de Paleontología, Facultad de Ciencias Geológicas, Universidad Complutense de Madrid, Madrid, Spain; 5 Departamento de Cambio Medioambiental, Instituto de Geociencias (UCM, CSIC), Madrid, Spain

**Keywords:** Fourier analysis, Dental morphology, Ecological characterization, Phenogram, Geometric morphometrics, Paleoecology, Rodentia, Mammalia, Miocene

## Abstract

Rodents are the most speciose group of mammals and display a great ecological diversity. Despite the greater amount of ecomorphological information compiled for extant rodent species, studies usually lack of morphological data on dentition, which has led to difficulty in directly utilizing existing ecomorphological data of extant rodents for paleoecological reconstruction because teeth are the most common or often the only micromammal fossils. Here, we infer the environmental ranges of extinct rodent genera by extracting habitat information from extant relatives and linking it to extinct taxa based on the phenogram of the cluster analysis, in which variables are derived from the principal component analysis on outline shape of the upper first molars. This phenotypic “bracketing” approach is particularly useful in the study of the fossil record of small mammals, which is mostly represented by isolated teeth. As a case study, we utilize extinct genera of murines and non-arvicoline cricetids, ranging from the Iberoccitanian latest middle Miocene to the Mio-Pliocene boundary, and compare our results thoroughly with previous paleoecological reconstructions inferred by different methods. The resultant phenogram shows a predominance of ubiquitous genera among the Miocene taxa, and the presence of a few forest specialists in the two rodent groups (Murinae and Cricetidae), along with the absence of open environment specialists in either group of rodents. This appears to be related to the absence of enduring grassland biomes in the Iberian Peninsula during the late Miocene. High consistency between our result and previous studies suggests that this phenotypic “bracketing” approach is a very useful tool.

## Introduction

Rodentia is the most species-rich group of mammals. The diversity of this group is linked to a high morphological disparity of dental features (Hunter & Jernvall, 1995). In general, dental morphology is functionally correlated with diet (Gómez Cano, Hernández Fernández & Álvarez-Sierra, 2013b; Kavanagh, Evans & Jernvall, 2007; Pineda-Munoz, Evans & Alroy, 2016) and therefore indirectly related to ecological features such as habitats (Auffray, Renaud & Claude, 2009; Blois & Hadly, 2009; Daams, Freudenthal & Van der Meulen, 1988; Martin, 2010). This relationship between shape and function in rodent teeth has been ultimately related to developmental processes (Gomes Rodrigues et al., 2013; Kavanagh, Evans & Jernvall, 2007).

Additionally, the paleontological record of rodents is mostly based on isolated teeth, given their hard mineralization. Teeth are particularly abundant in the fossil record and their highly informative morphology allows for the development of studies on evolutionary (e.g., Lazzari, Aguilar & Michaux, 2010) and ecological (e.g., Patnaik, 2011) patterns. Therefore, in order to work with fossil material it is essential to establish a methodology that utilizes isolated teeth.

Paleoecology of extinct micromammals has been inferred in several ways (Bonis et al., 2002; Domingo et al., 2012; Hernánde Fernández & Peláez-Campomanes, 2003a; Martín-Suárez, Freudenthal & Civis, 2001; Massol et al., 2011; Rodríguez, 2006). They are mostly community-based inferences, which required that fossil assemblage reflect or approximate the original community. On the other hand, using ecomorphological features for paleoecological inferences would be a taxon-oriented approach, rather than based on a community as a whole. Ecomorphological studies of extant rodent taxa have been conducted to show various degrees of morphological correlations with ecological and environmental parameters (Arregoitia, Fisher & Schweizer, 2017; Samuels, 2009; Samuels & Van Valkenburgh, 2008). This approach can provide paleoecological information for extinct taxa. However, these studies rarely include dental characters. Therefore we approached this dilemma and sought a way to fill this gap by building a database for M1 dental morphology of extant and extinct rodents. In this sense, geometric morphometric methodologies can quantify shape and size variation as well as the morphospace, which facilitates ecological and evolutionary inferences (Ledevin et al., 2016; Ledevin et al., 2010; Michaux, Chevret & Renaud, 2007; Renaud, Chevret & Michaux, 2007; Renaud et al., 1996; Samuels, 2009).

Following Hernánde Fernández & Peláez-Campomanes (2003b), we applied a phenotypic “bracketing” approach to geometric morphometric data of tooth morphology separately for the Murinae and non-arvicoline Cricetidae. Both groups are characterized by a specific occlusal pattern in their molars. Murines have three lingual cusps on the upper molars, giving a triserial cusp arrangement, which is characteristic to this subfamily. In relation to Cricetidae, due to the controversy associated to the arrangement of subfamilies among the extinct representatives (Fejfar, 1999; Hugueney, 1999; Kälin, 1999; Lindsay, 2008; Rummel, 1999), we here refer to them as Cricetidae *sensu lato* (s.l.) following López-Guerrero et al. (2017), which includes all the genera with typical “cricetid pattern” in the upper first molars (Schaub, 1925); this dental pattern shows four major cusps connected by crests, and one additional cusp at the anterior part of the first molar (anterocone). In this sense, representatives of Arvicolinae with a typical microtine pattern, in which cusps correspond to successive triangles in a varied number along the evolution of the group (Chaline et al., 1999), were excluded of our analysis.

The wide variety of environments inhabited by the extant genera and the great number of studies on extant and extinct taxa of rodents makes it possible to infer the palaeoecology of the extinct genera based on morphological similarities to extant taxa and to validate our approach by extensive comparisons with to previous works.

Although previous works on the geometric morphometrics of rodent molars describe some ecological preferences of these groups (Gómez Cano, Hernández Fernández & Álvarez Sierra, 2013b; McGuire, 2010; Renaud, Chevret & Michaux, 2007; Swiderski, Zelditch & Fink, 2000; Van Dam, 1997), the interest of our study is based on the use of a methodological approach that allows us for the first time to analyse the high morphological diversity within extant and extinct murine and cricetid rodents and link it with their biome preferences. The importance of the biome dimension lies in the integration of macroecological and macroevolutionary processes (Gómez Cano et al., 2013a; Hernánde Fernández & Vrba, 2005; Vrba, 1995).

## Material and Methods

### Extant samples

We chose the upper first molar (M1) for the ecomorphological study, because of its highly diagnostic features and abundance in the fossil record of muroids (e.g., Van Dam, 1997).

Based on the specimens available in museum collections and the literature, we compiled a total of 670 scaled pictures (see Supplementary Information 1 for specimen information and references) of right first upper molars for extant Murinae and non-arvicoline cricetids in our study, including extant subfamilies Cricetinae, Neotominae, Sigmodontinae and Tylomyinae (Musser & Carleton, 2005). These photos represent 107 of the 124 extant Murinae genera (around 86%), and in the case of Cricetidae (non-arvicoline) we included 85 of its 102 genera (around 83%) according to the taxonomic revision of (Musser & Carleton, 2005).

One of the authors (ARGC) took photographs of the tooth of extant taxa using a camera Nikon D300s and Nikon AF-S VR 105 mm f/2.8 IF-ED lens. These samples are housed at the American Museum of Natural History, New York (United States of America), the National Museum of Natural History, Washington DC (United States of America) and the Museum Nationale d’Histoire Naturelle, Paris (France).

### Climatic characterization of extant genera

In order to characterize environments potentially occupied by the extinct genera, we compiled the information for extant species of Murinae and Cricetidae s.l. under this study into a database, referring IUCN (2012) and Musser & Carleton (2005) for biogeographic distributions. Taking into account that different species of an extant genus may not have the same ecological parameters (e.g., *Peromyscus*), for each genus we summarized the climate zones inhabited by all its species. Although all the species of a genus may not have the same ecology, we consider that one genus summarizes all the ecomorphological variability of its species, due to the shared inheritance of aspects of morphology and habitat-specificity (Bobe & Eck, 2001; Greenacre & Vrba, 1984; Hernández Fernández & Vrba, 2006).

To determine the number of climate zones inhabited by one species, we adopted the cut-off of Hernánde Fernández (2001), in which the species is considered to occupy a climate zone if 15% or more of the geographical range of a species is situated within the climate zone. In addition, a species is also considered to occupy a specific climate zone when it inhabits 50% or more area in one climatic dominion. Here, a climatic dominion is defined as a continuous local climatic segment within a single climate zone. For instance, the winter rain and summer drought climate zone is present around the Mediterranean Sea and consists of several climatic dominions. In this climate zone, one climatic dominion covers the larger part of the Iberian Peninsula–South France, and other climatic dominion covers north-western Africa.

**Figure 1:**
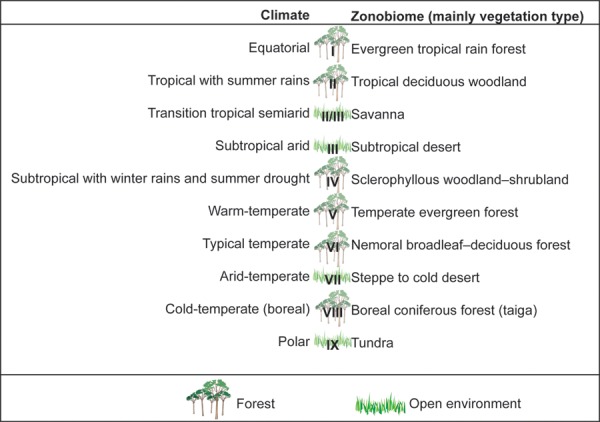
Bioclimatic climatic typology. Climatic typology used in this paper modified from Walter (1970) and its relationships with world vegetation types.

For the biome typology, we chose the climatic classification of Walter (1970) (Fig. 1), which has a simple nomenclature and combines climate and vegetation information. We added other classifications based on the characteristics of each biome, which simplifies the scheme, grouping taxa into three categories according the Walter’s biomes they inhabit: forest (I, II, IV, V, VI, VII), open environment (II/III, III, VII, IX) or ubiquitous if the taxon is present in both kinds of environments.

### Fossil samples

For extinct groups, we compiled a photographic database from the literature to include images of 11 extinct murine genera and 13 genera of extinct cricetids (Supplementary Information 1), which were characteristic for the Iberoccitanian region during the latest middle Miocene to the Mio-Pliocene boundary. We chose fossils from this region as a case study because this geographical area shows remarkable environmental differences from the rest of Europe today and during the Neogene (Gregor & Velitzelos, 1987; Kovar-Eder et al., 1996; López-Guerrero, 2006; Mai, 1989; Pickford & Morales, 1994; Van der Made, Morales & Montoya, 2006; Wolfe, 1985), and because previous studies have revealed environmental heterogeneity, with the existence of two mammalian bioprovinces (Álvarez-Sierra, García Moreno & López Martínez, 1985; Azanza et al., 1997; Daams et al., 1998; Gomez Cano et al., 2014; Heikinheimo et al., 2007; Morales et al., 1999).

The intensive sampling in the Iberoccitanian fossil sites and the great amount of detailed studies of these materials during the last decades (Sesé, 2006) made it possible to include 311 scaled pictures and drawings. These pictures represent all the extinct genera that have been from this region during the latest middle Miocene to the Mio-Pliocene boundary, including more than 90 species (see Appendix 1 for specimen information and references).

### Fourier analysis of the outline

We chose outline analysis to describe molar morphology because, besides being effective to describe the location of the tubercles characteristic of the molars, it is less sensitive than landmark analysis to modifications of the dental pattern due to wear (Renaud, 1999; Renaud et al., 1996). Furthermore, outline methods have been suggested to be useful tools for the analysis of biological shapes in the absence of sufficient homologous landmarks (Van Bocxlaer & Schultheiß, 2010).

The molar outline is defined as a two-dimensional projection of the molar viewed from the occlusal side. Following Renaud et al. (1996) these outlines were digitalized for each tooth as *x* and *y* coordinates of sixty-four points equally spaced along the tooth outline using TPSdig2 version 2.16 software (Rohlf, 2010). The starting point of each outline was defined at the maximum of curvature at the forepart of the tooth. In order to buffer the asymmetry between right and left molars within each individual, left molars were subjected to a mirror image and outlined as right molars (Renaud, 1999).

In order to analyze such *x* and *y* coordinates we applied an Elliptic Fourier Analysis (EFA) (Kuhl & Giardina, 1982) to the dataset using EFAwin software (Ferson, Rohlf & Koehn, 1985), which extracts Fourier coefficients from the original outline and normalizes these shape variables. By this method the complex outline could be described as a sum of trigonometric functions, named harmonics, of decreasing wavelength. Each harmonic is defined by four Fourier Coefficients (FCs), two for *x*-(A and B) and two for *y*-projections (C and D) (Deffontaine et al., 2009). The higher the rank of the Fourier harmonic, the more details of the outline it describes but it is expected that mathematical noise also increases (Renaud et al., 1996). Therefore, we used only Fourier coefficients up to the ninth harmonic following previous studies, which demonstrated that the effect of measurement error for upper molars was limited at this rank (Ledevin et al., 2010; Renaud, 1999). Finally, the first harmonic is proportional to the size of each specimen but its four coefficients (A1–D1) are constant because of the standardization and therefore will be omitted in the subsequent analyses (Renaud & Michaux, 2004; Renaud et al., 1996). Therefore, we retained eight harmonics, which represent the best compromise between measurement error and information content (Ledevin et al., 2010; Renaud, 1999). Thus we retained 32 FCs from these eight harmonics (i.e., A2–D9). Focusing studies on supraspecific taxa (genera, family etc.) can help to reveal their ecomorphological diversity and to understand the course of their adaptive evolution (Miljutin, 2011). Therefore, this work was carried out at the genus level, and each variable was calculated for every extant genus as the mean value of the included species (see Supplementary Information 2). Each set of Fourier coefficients was averaged per genus.

Finally, in order to represent accurate reconstructions of average outlines we used an inverse method of the elliptic Fourier transform (Kuhl & Giardina, 1982; Rohlf & Archie, 1984) as a support for visual interpretation of shape changes. These outlines based in the Elliptic Fourier Transformation method provides very accurate reconstructions because the inverse Fourier transform directly provide the Euclidean *xy*-coordinates of the reconstructed outline (Renaud & Michaux, 2003).

### The linking of extinct genera with extant ecological analogues

In order to relate the extinct genera with their modern ecological analogues, we computed phenograms using cluster analysis on the variables obtained from a principal component analyses (PCA) on the Fourier Coefficients. This limits the impact of covariance amongst variables on the subsequent clustering algorithm (Hempson, Archibald & Bond, 2015).

We first performed PCA independently for the two groups of rodents (murines and cricetids) to handle the obtained set of 32 Fourier Coefficients in the following cluster analysis. The PCA enables us to reduce redundant information among the Fourier Coefficients and to encompass the different patterns of shape variation within both groups. Finally, the principal components were then used to build a phenogram for each group. In order to reflect the statistical weight of each principal component we multiplied principal component scores by the fraction of the variance that each principal component explains. Then, these weighted scores were used to calculate a distance matrix based on Euclidean distances between group means. Finally, both cluster analyses (for murines and cricetids) were based on such distance matrices and used “paired-grouped” as clustering procedure, by means of the PAST software (Hammer, Harper & Ryan, 2001).

To infer the biome preference for the extinct genera we summarized the biomes of the extant genera with which they were closely linked in the cluster analysis. We chose this conservative assignation because it offers a glimpse of all the compatible environments for a particular morphology (Hernánde Fernández & Peláez-Campomanes, 2003b). Finally, exhaustive comparisons with previous works were conducted as a key in this study in order to validate that this ecomophological approach is valid.

## Results & Discussion

**Figure 2:**
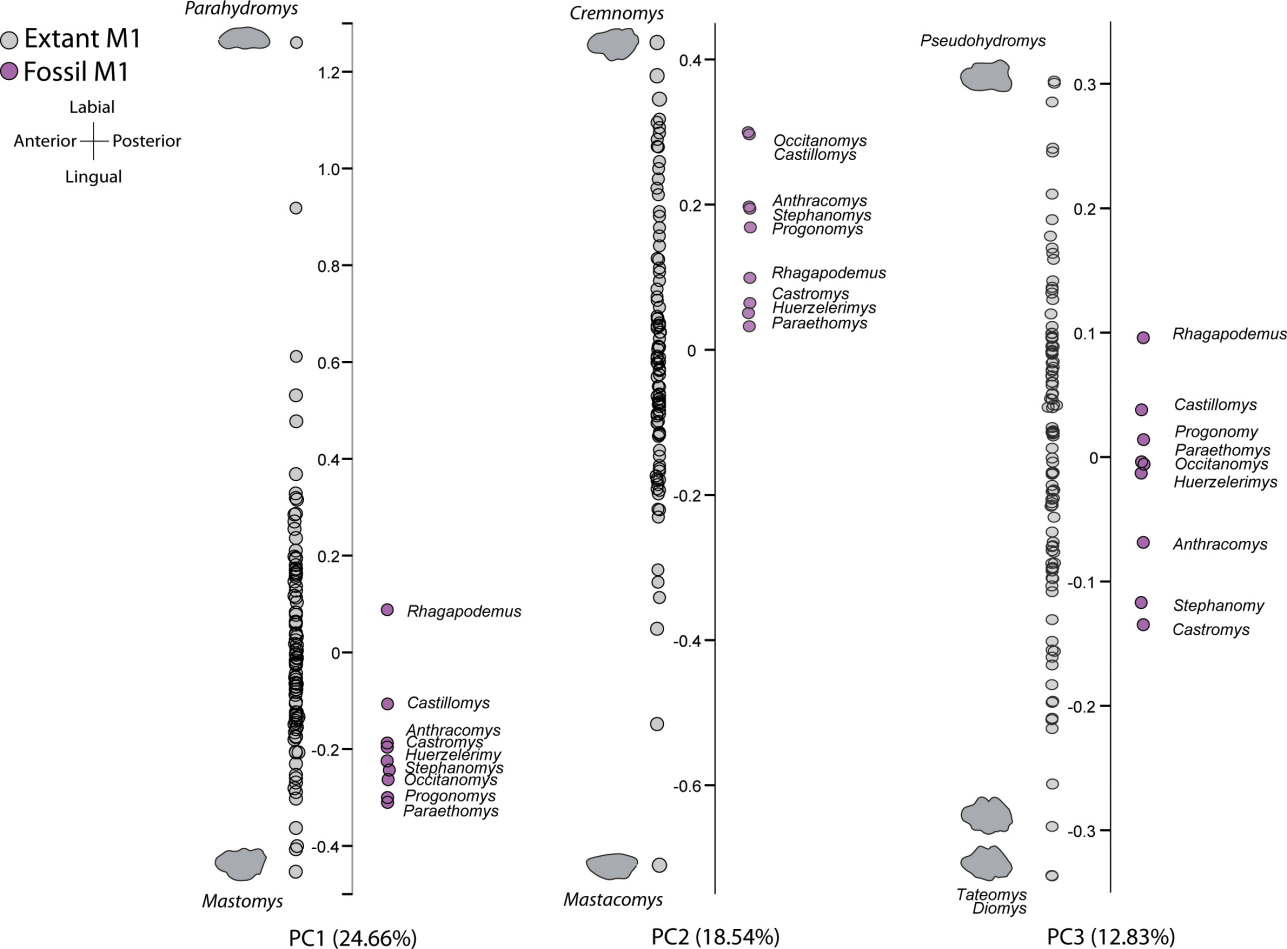
Graphs of principal components for murinae data. Ordination of murine genera in the morphospace defined by the first three principal components (PCs), based on elliptic Fourier coordinate datasets. Grey circles represent extant genera and purple circles represent extinct genera. The outlines of the extreme genera in each axis are shown.

**Figure 3:**
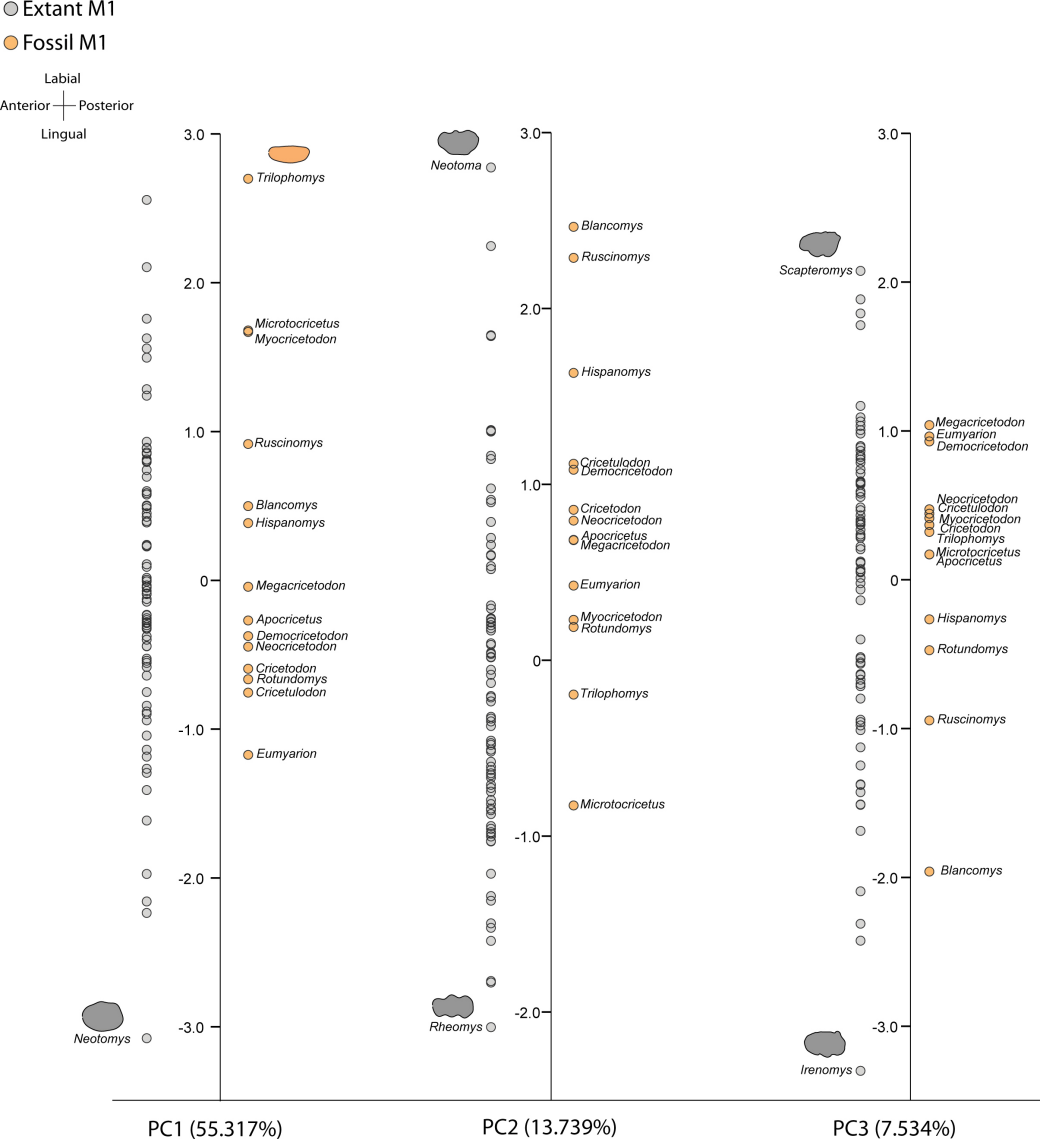
Graphs of principal components for cricetids data. Ordination of cricetid genera in the morphospace defined by the first three principal components (PCs), based on elliptic Fourier coordinate datasets. Grey circles represent extant genera and orange circles represent extinct genera. The outlines of the extreme genera in each axis are shown.

Although the basic pattern of major cusps is rather similar within each family, PCA results show that substantial difference in complexity affects the outline shape and allows us to differentiate several groups based on their morphological similarities (Figs. 2 and 3).

In the case of Murinae (Fig. 2), eight Principal Components were retained. The first principal component (PC1, 24.66% of the total variance) describes an axis from massive molars with the t1 clearly separated from the t2 cusp (negative values) to narrow molars with a weak lingual cusp (positive values). On the second principal component (PC2, 18.55%), the positive side of the axis is characterized by broad molars with the double t2 clearly independent from each other, while narrow molars with non-independent t2 fall on the negative side of this axis. Finally, the third component (12.83%) shows simple outlines symmetrical in the longitudinal axis (positive values) to more rounded molars with a robust t2 (negative values) and undulated outline where the main lingual cusps (t1 and t4) and labial cusp (t3, t6 and t9) are prominent and clearly identified in the outline.

The FC of Cricetids genera were reduced to six Principal Components. Reconstructed outlines for Cricetidae (Fig. 3) indicate that molars with narrow and elliptic molars are displayed on the positive end of the first principal component (PC1, 55.32% of the total variance), while more symmetrical round molars, which are more symmetrical are arranged at the negative end. The second axis (PC2, 13.74%) opposes massive molars (positive values) and molars with an undulating outline linked to disposition of main dental cusp (negative values). Finally, the third axis (PC3, 7.35%) opposes molars with the anterocone marked in the outline (positive values) and molars with rounded outline at the posterior side (negative values).

### Ecological characterization of Murinae

**Figure 4:**
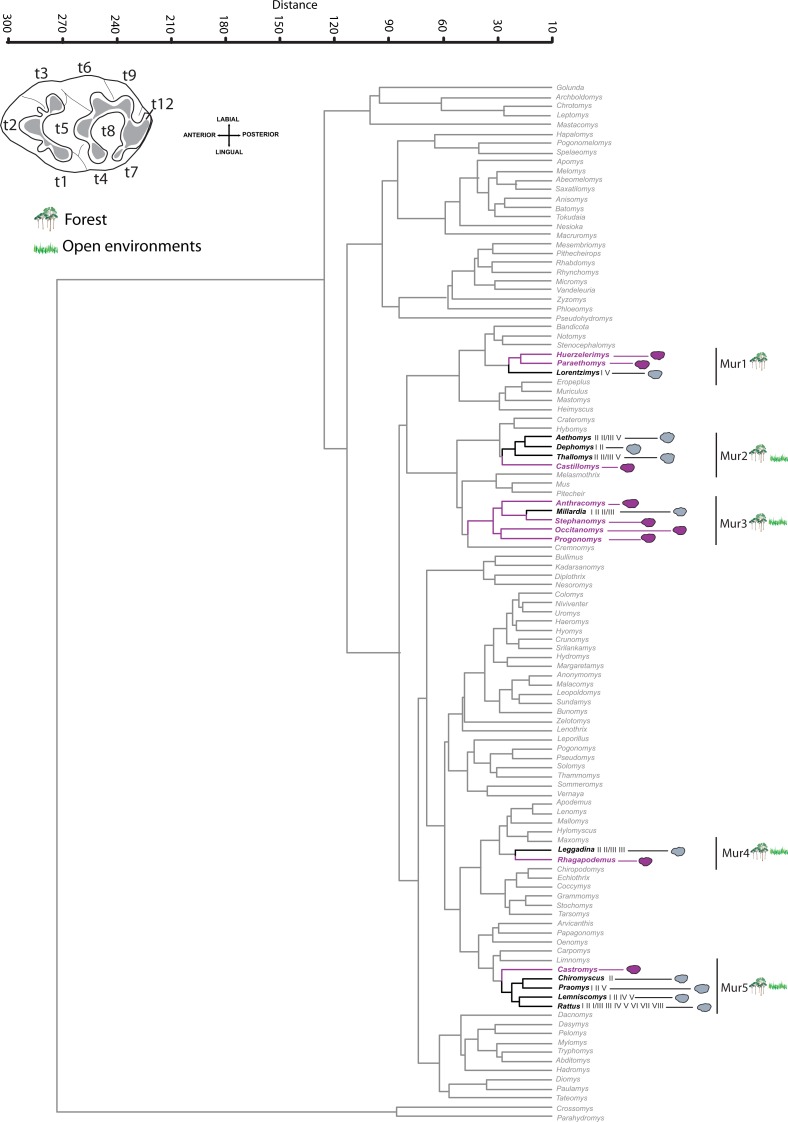
Murinae phenogram. Phenogram of the M1 outline similarity for studied extinct (purple) and the extant (grey) Murinae genera (Euclidean distance). The climate types (see Fig. 1) are shown for the extant genera, which were used for ecological inferences of extinct taxa. Terminology of the dental elements after Van der Weerd (1976). Outlines of genera within morphological groups containing extinct genera are represented. Taxonomy of extant genera according to Musser & Carleton (2005).

Based on the dendrogram of phenotypic similarities in the M1 outline obtained for Murinae (Fig. 4), we associated the extinct genera with five different morphological groups of extant taxa.

The first phenotypic group (Mur1) involves two extinct taxa (*Huerzelerimys* and *Paraethomys*) linked to the modern genus *Lorentzimys*, which inhabits humid forest biomes (I and V). These three genera have the t4 and t8 cusps connected by a weak crest whereas the t7 cusp is absent (Mein, Martín-Suárez & Agustí, 1993), which is reflected in the outline of the posterior side of the molar. The outlines of their molars are slightly narrower than those in group Mur2, and with the anterior part less differentiated than those in group Mur4. Whereas previous works based on communities and a morphological approach for the extinct taxa did not indicate specific ecological preferences for *Huerzelerimys,* humid and warm or non-seasonal environments have been inferred for *Paraethomys* (Hernánde Fernández & Peláez-Campomanes, 2003b; Martín-Suárez, Freudenthal & Civis, 2001), which are consistent with our results.

Group Mur2 involves the extinct *Castillomys*, which was linked to extant African taxa with preferences for both open and forested areas (generalist). *Castillomys* was bracketed by *Aethomys* (Musser & Carleton, 2005), *Thallomys*, and *Dephomys*. The former two genera are inhabitants of forested to open environments. *Aethomys* inhabits biomes II, II/III and V, and *Thallomys* commonly inhabits biome II but is also found in open environments (II/III) and subtropical desert (III). *Dephomys* is a rodent from tropical forests (biomes I and II). Regarding morphology, these genera present a t1 cusp, which is displaced backwards and is separated from the t2. Nevertheless, in these genera t2 is small, which gives a rounded external appearance of the outline (Fig. 4).

*Anthracomys, Occitanomys, Progonomys* and *Stephanomys* were grouped together (Mur3) and positioned closely in the cluster with the extant *Millardia*, which inhabits tropical biomes (both forest and open environments: biomes I, II and II/III). As occurred in group Mur2, in these five genera the t1 is displaced backwards and is separated from the t2. However, this feature is not developed in the same way in all these genera because this posterior position of t1 is more marked in some taxa than in other ones. Moreover this group has larger t2 than in group Mur2. These features generate slim outlines in the anterior area of the M1.

*Occitanomys* shows a great morphological similarity with *Stephanomys* (Freudenthal & Martín-Suárez, 1999; Van Dam, 1996; Van der Weerd, 1976). According to studies of paleocommunities and morphological features (Hernánde Fernández & Peláez-Campomanes, 2003b; Van Dam & Weltje, 1999), it appears that these two genera were associated with a mix of tropical woodlands and a mosaic environment with open and forest areas, which is indicative of the presence of a winter dry season. In addition, these genera show stephanodonty, which consists of the presence of broad molars with strong development of longitudinal crests connecting the transverse chevrons on the upper molars. This dental feature is moderate in *Occitanomys*, and more pronounced in *Stephanomys* (Renaud et al., 2005; Renaud & Van Dam, 2002; Uhlig, 2002). Stephanodonty has been linked to a general expansion of open environments, which has been noticed for the end of the Miocene in the Iberian region (Renaud et al., 2005). On the other hand, the upper first molar of *Anthracomys* has been described as possessing a high crown (Freudenthal & Martín-Suárez, 1999), which is also a feature that has been linked by Casanovas-Vilar et al. (2011) to extant murines from arid and open environments where grasses predominate. *Progonomys* which has a well-developed anterocentral cusp (Aplin & Helgen, 2010) is also grouped in this third set. In this case, Casanovas-Vilar & Agustí (2007) inferred open environments as the ecological preference for *Progonomys* based in synecological studies. This genus is the oldest murine recorded at the Iberoccitanian fossil sites and its entry, the *Progonomys* event, is classically related to the Vallesian Crisis where an expansion of relatively arid environments has been described (Agustí et al., 1997; Koufos, 2003). Finally, Gomes Rodrigues et al. (2013), showed that *Progonomys*, *Occitanomys* and *Stephanomys* had a similar herbivorous diet based in microwear analysis, which is consistent with the results of this paper. In summary, the environmental envelopes described by previous authors for the murine included in this third set agree with our ecological inference, a wide range of habitats from tropical to relatively arid environments, which are characterized by a mosaic habitat of forest and herbaceous plants.

*Rhagapodemus* was linked to the extant genus *Leggadina* (Mur4), which has been found in tropical woodlands, savannas and desert areas (biomes II, II/III and III). The absence of longitudinal connections between the cusps makes the molar structure of extinct *Rhagapodemus* consistent with that of *Leggadina*. Furthermore the labial edge of the outlines is less sinuous than in the other groups of murines in our study. Previous works have provided inconsistent inference, linking *Rhagapodemus* records with open or arid environments in the Iberian Peninsula but also in other sites from Italy and Turkey (Casanovas-Vilar et al., 2011; Jiménez-Moreno et al., 2015; Van der Made, Morales & Montoya, 2006) or with forested environments (Hernánde Fernández & Peláez-Campomanes, 2003b). Our results suggest that *Rhagapodemus* was a generalist taxon that could inhabit both open and forest ecosystems, which could be consistent to both interpretations of previous studies.

Finally, the extinct genus *Castromys* was linked to four extant genera (Mur5). *Lemniscomys* is a member of an African clade (Musser & Carleton, 2005) and inhabits forested biomes (I, II, IV and V). *Praomys* occurs primarily in forest and woodland habitats (biomes I, II and V), although extinct representatives of this genus were linked to open, dry environments (Geraads, 1995; Geraads et al., 1998). The murine *Chiromyscus* is an arboreal rodent that inhabits tropical deciduous woodlands (specialist), but it also occurs in mosaic scrub areas as extensions of forest and other degraded habitats (Aplin & Lunde, 2008). Finally, *Castromys* is also linked to the widespread genus *Rattus*, which involves a great disparity of morphologies and inhabits the majority of biomes (I, II, II/III, III, IV, V, VI, VII and VIII). These taxa have molars with broad cusps and without connection between t6 and t9 (although this characteristic is variable in some species of *Rattus*), which affect mostly the posterolabial side of the outline (Freudenthal & Martín-Suárez, 1999; Martín-Suárez & Freudenthal, 1994; Wessels, 2009). In this case, the molars are broader in relation to their length than in the others groups. Martín-Suárez, Freudenthal & Civis (2001) assigned cool environmental preferences for *Castromys* based in a correlation between localities of Crevillente (South-eastern of Iberian Peninsula) and the eustatic sea level curve; the presence of *Castromys* in Crevillente localities is associated with eustatic minima and, therefore, was assigned a cold/dry preference. However, Van Dam & Weltje (1999) assumed preferences for intermediate temperatures (subtropical), as inferred by modelling of Miocene rodent palaeocommunities. Our results do not indicate any specific environmental preferences for *Castromys*, which should be regarded as a generalist rodent.

### Ecological characterization of Cricetidae

**Figure 5:**
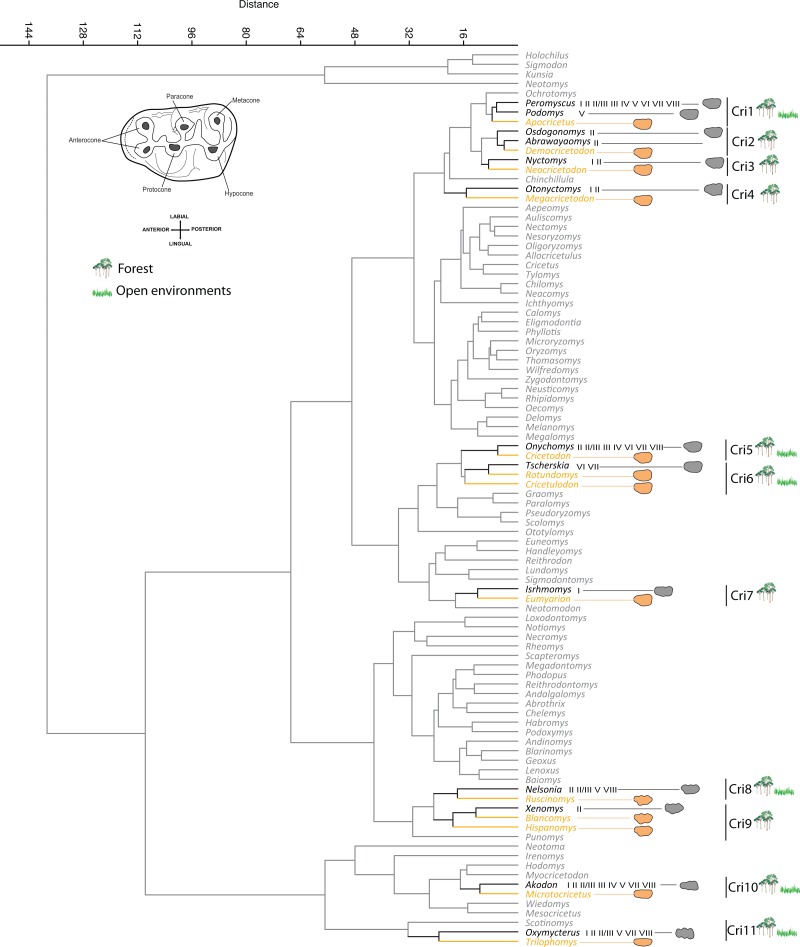
Cricetids phenogram. M1 morphological similarity cluster of studied extinct (yellow) and the extant (grey) Cricetidae genera (Euclidean distance). The climate types (see Fig. 1) are shown for the extant genera, which were used for ecological inferences of extinct taxa. Morphology of the occlusal surface is shown; terminology of the dental elements after Oliver & Peláez-Campomanes (2013). Outlines of genera within morphological groups containing extinct genera are represented. Taxonomy of extant genera according to Musser & Carleton (2005).

The genera of extinct Cricetidae included in this analysis can be differentiated in eleven morphological groups (Fig. 5). The first three groups form a large agglomerative group of genera, which have similar outlines with less marked differences among them. These genera show clear distinction between the labial and lingual edges, which generates a longitudinally asymmetric molar.

The first group (Cri1) includes the extinct genera *Apocricetus* and the extant *Podomys* and *Peromyscus*. These genera show a prominent metacone (posterolabial cusp), which characteristically generates the rounded and protruding outline in the posterolabial region. *Podomys* is considered a forest specialist (Duesser & Shugar Jr, 1978; Gibbes & Barrett, 2011) of temperate environments (biome V). However, *Peromyscus* is the most speciose genus of the Neotominae subfamily, and its diversity is linked to a wide range of different biomes inhabited by the species of this genus. Several previous works, based on studies of environmental inferences by micromammalian communities (García-Alix, 2006; García-Alix et al., 2008; Minwer-Barakat, 2005), associated some *Apocricetus* species with open/herbaceous and warm environments. Other studies based on community approximation and ecomorphological studies (Daams, Freudenthal & Van der Meulen, 1988; Hershkovitz, 1962; Martín-Suárez, Freudenthal & Civis, 2001; Van Dam & Weltje, 1999), link *Apocricetus* to warm and humid conditions in forest environments. Our results are congruent with previous studies and showed *Apocricetus* as a generalist, which should be linked to both open and forested environments.

*Democricetodon* was clustered in the second morphological group (Cri2) together with the extant genera *Osdogonomys* and *Abrawayaomys*. Despite the high similarity in dental features between *Megacricetodon* and *Democricetodon* (Wessels, 2009), in general the genus *Democricetodon* differs from the genus *Megacricetodon* in having a relatively shorter and broader outline (Fejfar et al., 2011). Although our results are congruent with the high degree of similarity between *Democricetodon* and *Megacricetodon*, the differences between these extinct genera are evident in the outlines and link *Democricetodon* with extant genera that possess broader molars and the anterolingual side less marked in the outline than in *Megacricetodon*. The dental pattern described for *Democricetodon* as low-crowned bunodont molars could be linked with wet environments (Hershkovitz, 1962). Nevertheless, although increase in morphological variability within *Democricetodon* teeth has been correlated to periods of decreasing temperature (Peláez-Campomanes, Hernández-Ballarín & Oliver, 2015), some authors have indicated that changes in humidity across the Iberian Miocene did not affect the presence of *Democricetodon* (Freudenthal & Mein, 1989). These indications could be coherent with the environment for *Osdogonomys* and *Abrawayaomys*, which are described as tropical deciduous woodland inhabitants (biome II). This kind of forest is characterized by the seasonal alternation of winter-dry and summer-wet periods.

In the third group (Cri3) the extinct genus *Neocricetodon* clusters with the extant genus *Nyctomys*. Despite a high degree of similarities in outline with the previous group, these two genera show narrower molars than those in groups Cri1 and Cri2, and the metacone cusp is less prominent, which is seen in our results as a straighter labial outline. *Nyctomys* has been described as an arboreal or semi-arboreal rodent, which inhabits tropical forests (I and II) and has low-crowned molars (Carleton, 1980; Corley, Ordóñez Garza & Rogers, 2011; Hunt, Morris & Best, 2004). Even if *Neocricetodon* has been previously described as eurytopic (Van Dam & Weltje, 1999), it is commonly linked to forested environments (Colombero et al., 2017), which is congruent with our results that showed *Neocricetodon* is a forested specialist.

*Megacricetodon* is linked to the extant genus *Otonyctomys* in the group Cri4; both genera show a distinct lingual outline due to the development and position of the anterocone, which makes the anterior part of the tooth limited and marked in the outline with respect to the rest of the main cusps. The brachyodont and mesodont molars with bunodont dental pattern described in *Megacricetodon* species (Fejfar et al., 2011; Van Dam & Weltje, 1999) have been previously associated with humid environments (Daams, Freudenthal & Van der Meulen, 1988), which is coherent with the biomes inhabited by *Otonyctomys* in the Yucatan Peninsula (I and II).

In the fifth set, Cri5, *Cricetodon* is grouped with the extant genus *Onychomys*. Both genera show a relatively similar outline to the previous three groups, sharing an asymmetrical outline, due to the narrowing of the anterior edge of the tooth. Nevertheless, the outline that corresponds to the anterocone is, in proportion, more slender compared with the previous groups. The North American rodent *Onychomys* inhabits several biomes (II, II/III, III, IV, VI, VII, VIII), most of them characterized by the presence of an annual dry season. Although for some species of *Cricetodon* Rummel (1999) indicated preferences for forested areas, other authors suggested drier environmental preferences for this genus based on high-crowned molars (De Bruijn & Ünay, 1996; Van Dam & Weltje, 1999). Our results integrate the conflict into a generalist characterization for the genus.

*Rotundomys* and *Cricetulodon,* which are closely related genera (López-Antoñanzas, Peláez-Campomanes & Álvarez Sierra, 2014), were clustered together in the group Cri6 with the extant *Tscherskia*, which is associated with temperate biomes (VI and VII) inhabiting both forest and open environments. All these genera have an elliptic outline and share some dental features. For example, the lophodont tooth pattern described for *Rotundomys* (Fejfar et al., 2011; Kälin, 1999; Van Dam & Weltje, 1999) indicates food preferences on fibrous plant parts (Casanovas-Vilar & Agustí, 2007). This food habit also has been described for *Tscherskia,* which shares low-crowned molars (Nechay, 2000; Neumann et al., 2006).

In the group Cri7, the extinct genera *Eumyarion* was placed morphologically close to the extant *Isthmomys*. These genera have asymmetrical molars similar to the ones in the first four groups (Cri1 to Cri4). Nevertheless, Cri7 taxa differ from them in the posterior side where they present a straight outline, which could be related to the alignment between the metacone and the hypocone. The two species included in the genus *Isthmomys* have a small geographic range in Panama limited to equatorial rain forest areas. Therefore, our results are consistent with Kälin (1999), who assumed similar forest conditions for *Eumyarion*, based on brachyodonty.

In the morphological groups Cri8 and Cri9 we found molars with a marked sinuous outline both labially and lingually, which is linked to the high crown (hypsodonty) and the transverse alignment of each cusp. In the group Cri8 *Ruscinomys* is linked to the extant genera *Nelsonia*. *Nelsonia* occurs in both open and forest biomes (II, II/III, V, VIII), while it has been described within these biomes as inhabitant of cool cliffs, rim rock and other rocky situations found in canyon bottoms (Hooper, 1954). Although, *Ruscinomys* has been considered as inhabitant of open/herbaceous environments (García-Alix et al., 2008; Van Dam & Weltje, 1999). We mostly agree with Martín-Suárez, Freudenthal & Civis (2001) and Hernánde Fernández & Peláez-Campomanes (2003b) considering this genus as an ecological generalist.

The group Cri9 showed the extinct genera *Hispanomys* and *Blancomys* linked to the extant *genus Xenomys*, which inhabits dry tropical woodlands (biome II). While some authors considered *Hispanomys* as inhabitants of open/herbaceous environments (De Bruijn et al., 1993; García-Alix et al., 2008; Van Dam & Weltje, 1999), other studies indicated more generalist ecological preferences for this genus (Martín-Suárez, Freudenthal & Civis, 2001; Hernánde Fernández & Peláez-Campomanes, 2003b). In the case of *Blancomys*, studies based on morphological and synecological approximations had slightly different outcomes; whereas Hernánde Fernández & Peláez-Campomanes (2003b) indicated preference for dry habitats, García-Alix et al. (2008) pointed to preferences for open and probably fresh environments. Most of these interpretations might be encompassed by our results, since biome II is present in tropical environments with strong hydric seasonality.

The last two groups (Cri10 and Cri11) involved separately *Microtocricetus* and *Trilophomys*, which show similar outlines with particularly narrow molars (Fig. 5). The outlines of these groups are particularly symmetrical; that is because the outline at the bi-lobed anterocone is very symmetrical, broader than in the previous groups, and reduced in relation to the posterior side.

The extinct *Microtocricetus* is linked to the extant genus *Akodon* (Cri10), which inhabits both forested and open biomes (I, II, II/III, III, IV, V, VII and VIII). The outlines of these two genera are very symmetrical with a flat occlusal surface and a rectangular shape (János & Kókay, 2010; Wahlert, 1984). The molars of *Microtocricetus* have been described as mesodont, trending toward a prismatic dental pattern (Fejfar et al., 2011), and this genus was linked to open environments (Bachmayer & Wilson, 1985).

The extinct genus *Trilophomys* forms the group Cri11 with the extant genera *Oxymycterus*, which is also present in a wide range of biomes (I, II, II/III, V, VII and VIII). In general, the molars of these rodents show a trend in which cusps are opposed and begin to fuse into transverse lophs (Wahlert, 1984). The outline is symmetrical as in the previous group, although in the case of *Trilophomys* the anterior side is a little more differentiated and this area is slightly narrower when compared with the fifth group. The molars of *Trilophomys* have been described as mesodont and hypsodont and, as in the extant taxa, have a prismatic dental pattern (Fejfar et al., 2011), which is related to open environments where grasslands are dominant. Previous palaeoecological inferences linked this extinct genus to open environments (García-Alix et al., 2008; Hernánde Fernández & Peláez-Campomanes, 2003b). However, although most biomes inhabited by the extant genus of this morphological group are characterized by the occurrence of a dry season and several species of *Oxymycterus* are found in high altitudes with cold and open landscapes (Hershkovitz, 1962), they show very generalist ecology and occur in both forest and grassland biome environments. In this sense, our results appear to expand the ecological preferences of *Trilophomys*.

### Final remarks

We note the remarkable absence of open environment specialists (Figs. 4 and 5) in both groups of rodents (Cricetidae and Murinae). In summary, our results show in the two rodent groups a predominance of ubiquitous genera among the Miocene taxa, and the presence of a few forest specialists.

This absence of open environments specialists is coherent with the fact that there is no isotopic evidence of C_4_ plant-dominated habitats in the Iberoccitanian region during the Miocene (Domingo et al., 2013), which is related to the absence of long-term C_4_-dominated open grasslands in favour of forests and open woodlands (Domingo et al., 2009).

On the other hand, the dominance in our results of generalist taxa could be related to their tendency to survive in unstable environments with changing conditions (Cantalapiedra, Hernández Fernández & Morales, 2011; Gomez Cano et al., 2013; Hernánde Fernández & Vrba, 2005; Moreno Bofarull et al., 2008; Van der Meulen, Peláez-Campomanes & Levin, 2005; Vrba, 1980; Vrba, 1987), as the ones that could be found in the transition zone between tropical and temperate biogeographic regions that was the Iberian Peninsula during the Neogene (Pickford & Morales, 1994).

Our study was based in the late Miocene Iberoccitanian extinct rodents. Many of the studied genera had species living outside the Iberoccitanian region or in additional time intervals not considered here (e.g., *Megacricetodon* and *Democricetodon*, have been registered in Africa and Asia, as well as in earlier time intervals), which might increase the total morphological range of these genera. Consequently, the associated extant genera could slightly change in some cases, particularly in those genera that have very few species in the Iberoccitanian region during the late Miocene (e.g., *Eumyarion* with only two species considered in this study, but much more diverse in Central Europe and even Asia during the Oligocene and Miocene). Therefore, although our results are a good characterization of extinct rodent genera from the South-western Europe during the Late Miocene, they must be taken carefully when extrapolated to other time intervals or biogeographical regions.

## Conclusions

Geometric morphometric comparison of the first upper molars in several extant and extinct rodent taxa allowed the inference of ecological preferences based on dental morphology at the genus level. This work set this analysis of geometric morphometric as a very effective methodology for eco-morphological inferences by comparing with previous works using different methods. This method is particularly useful in the study of the fossil record of small mammals, which is mostly represented by isolated teeth.

Particularly, the morphological database of upper molar outlines of extant taxa compiled in this work is a successful tool for the development of ecomorphological analyses of the rodent fossil record.

Finally, the ecological characterization established in this work will be indispensable for the future development of palaeoecological and palaeoclimatic studies, which could help further explain the ecological diversity of the Iberoccitanian region during the Miocene.

##  Supplemental Information

10.7717/peerj.3646/supp-1 Supplemental Information 1 References for samples used in this study Collection number and references of the extant and extinct murine rodent samples used in this work.

10.7717/peerj.3646/supp-2 Supplemental Information 2 Table with the calculated dataset of FC for each extant and extinct genus considered in this works after EFA Values of the Fourier Components for each extant and extinct genus considered in this work after Elliptic Fourier Analysis.

## Additional Information and Declarations

### Data Availability

The following information was supplied regarding data availability: The raw data has been supplied as Supplementary File.
