# Supplementary material for: Ecomorphological characterization of murines and non-arvicoline cricetids (Rodentia) from south-western Europe since the latest Middle Miocene to the Mio-Pliocene boundary (MN 7/8–MN13)
Source: PeerJ. 2017 Sep 25;5:e3646. doi: 10.7717/peerj.3646 (PMC5619236; doi:10.7717/peerj.3646)
Supplement: Supplemental Information 1 — Collection number and references of the extant and extinct murine rodent samples used in this work. [file peerj-05-3646-s001.pdf]

Collection number and references of the extant and extinct rodent used in this work. *indicates the specimens for which we take the photograph in the Museum collections (Musée National d'Histoire Naturelle, Paris; American Museum of Natural History, New York; National Museum of Natural History, Washington DC).

Family	Subfamily	*Species*	References	Fossil site	Specimen ID	Extant/ Extinct
Muridae	Murinae	*Abditomys sp.*	Musser & Headley 1992		USNM 357244	Extant
Muridae	Murinae	*Abelomelomys sevia*	Musser & Lunde 2009		AMNH 192119	Extant
Muridae	Murinae	*Acomys dimidiatus*	*		2001 3	Extant
Muridae	Murinae	*Aethomys hindei*	*		MNHN 1999 177	Extant
Muridae	Murinae	*Aethomys namanquesis*	*		MNHN 1964 57	Extant
Muridae	Murinae	*Aethomys nigeriae*	*		MNHN 1996 2239	Extant
Muridae	Murinae	*Anisomys imitator*	Missonne 1969		BMNH 471310	Extant
Muridae	Murinae	*Anonymomys mindorensis*	Musser 1981		FMNH 87597	Extant
Muridae	Murinae	*Anonymomys sp.*	Musser & Headley 1992		FMNH 87598	Extant
Muridae	Murinae	*Apodemus agrarius*	*		MNHN BL6 1134	Extant
Muridae	Murinae	*Apodemus sylvaticus*	*		MNHN 1994 2667	Extant
Muridae	Murinae	*Apodemus sylvaticus*	*		MNHN 1994 2668	Extant
Muridae	Murinae	*Apodemus sylvaticus*	*		MNHN 1994 2671	Extant
Muridae	Murinae	*Apodemus sylvaticus*	*		MNHN 1994 2679	Extant
Muridae	Murinae	*Apodemus sylvaticus*	*		MNHN 1994 2681	Extant
Muridae	Murinae	*Apodemus sylvaticus*	*		MNHN 1994 945	Extant
Muridae	Murinae	*Apomys sp.*	Musser & Headley 1992		12971 CLM1-3	Extant

Collection number and references of the extant and extinct rodent used in this work. *indicates the specimens for which we take the photograph in the Museum collections (Musée National d'Histoire Naturelle, Paris; American Museum of Natural History, New York; National Museum of Natural History, Washington DC).

**Family**	**Subfamily**	***Species***	**References**	**Fossil site**	**Specimen ID**	**Extant/ Extinct**
Muridae	Murinae	*Archboldomys sp.*	Musser & Headley 1992		FMNH 95122	Extant
Muridae	Murinae	*Arvicanthis niloticus*	*		MNHN 2007 217	Extant
Muridae	Murinae	*Bandicota indica*	*		MNHN 1986 366	Extant
Muridae	Murinae	*Batomys sp.*	Musser & Headley 1992		FMNH 74853	Extant
Muridae	Murinae	*Bullimus sp.*	Musser & Headley 1992		DMNH 4173	Extant
Muridae	Murinae	*Bunomys chysocomus*	Musser & Durden, 1991		MZB 12185	Extant
Muridae	Murinae	*Bunomys prolatus*	Musser & Durden, 1991		MZB 12190	Extant
Muridae	Murinae	*Carpomys sp.*	Musser & Headley 1992		USNM 102549	Extant
Muridae	Murinae	*Castillomys gracilis*	Adrover, 1986		ALD 116	Extant
Muridae	Murinae	*Castillomys gracilis*	Adrover, 1986		VA1 185	Extant
Muridae	Murinae	*Castromys inflatus*	Martín-Suárez & Freudenthal, 1994		RGM 413 759	Extant
Muridae	Murinae	*Castromys littoralis*	Freudenthal & Martín-Suárez, 1999		RGM 413 092	Extant
Muridae	Murinae	*Castromys littoralis*	Martín-Suárez & Freudenthal, 1994		RGM 413 103	Extant

Collection number and references of the extant and extinct rodent used in this work. *indicates the specimens for which we take the photograph in the Museum collections (Musée National d'Histoire Naturelle, Paris; American Museum of Natural History, New York; National Museum of Natural History, Washington DC).

Family	Subfamily	*Species*	References	Fossil site	Specimen ID	Extant/ Extinct
Muridae	Murinae	*Chiromyscus chiropus*	*		1929 298	Extant
Muridae	Murinae	*Chiromyscus chiropus*	Musser 1981		USNM 321507	Extant
Muridae	Murinae	*Chiropodomys calamianensis*	Tate, 1936		AM109999	Extant
Muridae	Murinae	*Chiropodomys gliroides*	*		MNHN 1981 283	Extant
Muridae	Murinae	*Chrotomys sp.*	Musser & Headley 1992		AMNH185138	Extant
Muridae	Murinae	*Chrotomys sp.*	Musser & Headley 1992		FMNH 62279	Extant
Muridae	Murinae	*Chrotomys sp.*	Musser & Headley 1992		MMNH 12972	Extant
Muridae	Murinae	*Coccymys ruemmleri*	Musser & Lunde 2009		AMNH 192737	Extant
Muridae	Murinae	*Colomys goslingi*	Misonne 1969		MRAC 21953	Extant
Muridae	Murinae	*Crateromys sehadenbergi*	*		MNHN 1897 414	Extant
Muridae	Murinae	*Crateromys sp.*	Musser & Headley 1992		USNM 102546	Extant
Muridae	Murinae	*Cremnomys sp.*	*		1962 1029	Extant
Muridae	Murinae	*Crossomys moncktoni*	Helgen 2005			Extant
Muridae	Murinae	*Crunomys melanius*	Musser et al., 2002		AMNH 224316	Extant
Muridae	Murinae	*Crunomys sp.*	Musser & Headley 1992		AMNH 242102	Extant
Muridae	Murinae	*Dacnomys millardi*	Musser 1981		FMNH 114175	Extant

Collection number and references of the extant and extinct rodent used in this work. *indicates the specimens for which we take the photograph in the Museum collections (Musée National d'Histoire Naturelle, Paris; American Museum of Natural History, New York; National Museum of Natural History, Washington DC).

**Family**	**Subfamily**	***Species***	**References**	**Fossil site**	**Specimen ID**	**Extant/ Extinct**
Muridae	Murinae	*Dacnomys millardi*	Tate1936		AM103253	Extant
Muridae	Murinae	*Dasymys incomitus*	*		MNHN 1970 563 3977	Extant
Muridae	Murinae	*Dasymys incomptus*	*		MNHN 1982 1037	Extant
Muridae	Murinae	*Dasymys rufulus*	*		MNHN 2004 1091	Extant
Muridae	Murinae	*Dephomys defua*	*		1991 2102	Extant
Muridae	Murinae	*Dephomys defusa*	*		1991 2101	Extant
Muridae	Murinae	*Dephomys eburnea*	*		1997 2205	Extant
Muridae	Murinae	*Diomys crumpi*	Missonne 1969		BMNH 47227	Extant
Muridae	Murinae	*Diplothrix legata*	Wang et al 2010			Extant
Muridae	Murinae	*Echiothrix leucura*	Musser, 1990		AMNH 225681	Extant
Muridae	Murinae	*Echiothrix leucura*	Tate, 1936		101248	Extant
Muridae	Murinae	*Eropeplus canus*	Misonne 1969		USNM 3336	Extant
Muridae	Murinae	*Eropeplus canus*	Tate, 1936		AM2601	Extant
Muridae	Murinae	*Golunda ellioti*	*		MNHN 1999 17	Extant
Muridae	Murinae	*Grammomys butingi*	*		MNHN 1980 270	Extant
Muridae	Murinae	*Grammomys caniceps*	*		MNHN 1986 1091	Extant
Muridae	Murinae	*Grammomys dolichirus*	*		MNHN 2005 6175	Extant
Muridae	Murinae	*Grammomys dolichurus*	*		MNHN 2006 602	Extant
Muridae	Murinae	*Grammomys dolichurus*	*		MNHN 2006 603	Extant
Muridae	Murinae	*Grammomys gazellae*	*		MNHN 1982 528	Extant
Muridae	Murinae	*Grammomys macmillani*	*		MNHN 2000 28	Extant
Muridae	Murinae	*Grammomys macmillani*	*		MNHN 2000 29	Extant

Collection number and references of the extant and extinct rodent used in this work. *indicates the specimens for which we take the photograph in the Museum collections (Musée National d'Histoire Naturelle, Paris; American Museum of Natural History, New York; National Museum of Natural History, Washington DC).

**Family**	**Subfamily**	***Species***	**References**	**Fossil site**	**Specimen ID**	**Extant/ Extinct**
Muridae	Murinae	*Grammomys rutilans*	*		MNHN 1966 244	Extant
Muridae	Murinae	*Grammomys rutilans*	*		MNHN 1996 2221	Extant
Muridae	Murinae	*Grammomys sudaster*	*		MNHN 1996 680	Extant
Muridae	Murinae	*Gramonomys dolichirus*	*		6175 136	Extant
Muridae	Murinae	*Hadromys humei*	Misonne 1969		BMNH 217871	Extant
Muridae	Murinae	*Hadromys humei*	Musser, 1987		FMNH 76567	Extant
Muridae	Murinae	*Haeromys minahassae*	Musser, 1990		AMNH 226048	Extant
Muridae	Murinae	*Hapalomys longicaudatus*	*		MNHN 1977 248	Extant
Muridae	Murinae	*Hapalomys longicaudatus*	Tate, 1936		AM54754	Extant
Muridae	Murinae	*Heimyscus fumosus*	*		MNHN 2008 244	Extant
Muridae	Murinae	*Hybomys trivirgatus*	*		MNHN 1979 420	Extant
Muridae	Murinae	*Hybomys univittatus*	*		MNHN 1963 210	Extant
Muridae	Murinae	*Hydromys habbema*	Musser & Headley 1992		AMNH 110057	Extant
Muridae	Murinae	*Hydromys hussoni*	Helgen, 2005			Extant
Muridae	Murinae	*Hylomyscus waltervercheyeni*	*		MNHN 2007 754	Extant
Muridae	Murinae	*Hyomys alleni*	*		MNHN 1991 2128	Extant
Muridae	Murinae	*Hyomys meeki*	Tate, 1936		AM79781	Extant
Muridae	Murinae	*Kadarsanomys sodyi*	Musser, 1981		RMNH14103	Extant
Muridae	Murinae	*Laphuromys aquilus*	*		1912 27	Extant
Muridae	Murinae	*Laphuromys aquilus*	*		1912 27	Extant
Muridae	Murinae	*Leggadina delicatulus*	Misonne 1969		BMNH 35742	Extant

Collection number and references of the extant and extinct rodent used in this work. *indicates the specimens for which we take the photograph in the Museum collections (Musée National d'Histoire Naturelle, Paris; American Museum of Natural History, New York; National Museum of Natural History, Washington DC).

**Family**	**Subfamily**	***Species***	**References**	**Fossil site**	**Specimen ID**	**Extant/ Extinct**
Muridae	Murinae	*Leggadina hermannsburgensis*	*		MNHN 1971 543	Extant
Muridae	Murinae	*Leggadina lakedownensis*	Cooper et al., 2003		M16906	Extant
Muridae	Murinae	*Lemniscomys barbarus*	*		MNHN 1975 117	Extant
Muridae	Murinae	*Lemniscomys bellieri*	*		MNHN 2006 208	Extant
Muridae	Murinae	*Lemniscomys griselda*	*		MNHN 1889 190	Extant
Muridae	Murinae	*Lemniscomys linulus*	*		MNHN 2004 1063	Extant
Muridae	Murinae	*Lemniscomys macculus*	*		MNHN 1992 1549	Extant
Muridae	Murinae	*Lemniscomys rosalia*	*		MNHN 1996 569	Extant
Muridae	Murinae	*Lemniscomys zebra*	*		MNHN 2004 135	Extant
Muridae	Murinae	*Lenomys meyeri*	Misonne 1969		BMNH 991019	Extant
Muridae	Murinae	*Lenomys meyeri*	Tate, 1936		AM1011125	Extant
Muridae	Murinae	*Lenothrix canus*	*		MNHN 1977 246	Extant
Muridae	Murinae	*Leopoldomys sabanus*	*		MNHN 1977 377	Extant
Muridae	Murinae	*Leporillus apicalis*	*		MNHN 1882 2173	Extant
Muridae	Murinae	*Leptomys sp.*	Musser & Headley 1992		AMNH 105793	Extant
Muridae	Murinae	*Limnomys 144621*	Musser & Headley 1992		144621	Extant
Muridae	Murinae	*Limnomys 5956*	Musser & Headley 1992		5956	Extant
Muridae	Murinae	*Limnomys sibuanus*	Musser 1981		USNM 144621	Extant
Muridae	Murinae	*Lorentzimys nouhuysi*	Misonne 1969		BMNH 53329	Extant

Collection number and references of the extant and extinct rodent used in this work. *indicates the specimens for which we take the photograph in the Museum collections (Musée National d'Histoire Naturelle, Paris; American Museum of Natural History, New York; National Museum of Natural History, Washington DC).

**Family**	**Subfamily**	***Species***	**References**	**Fossil site**	**Specimen ID**	**Extant/ Extinct**
Muridae	Murinae	*Malacomys edwardsi*	*		MNHN 2008 86	Extant
Muridae	Murinae	*Malacomys longipes*	*		MNHN 1995 1217	Extant
Muridae	Murinae	*Malacomys lukolelae*	*		MNHN 1991 869	Extant
Muridae	Murinae	*Mallomys rothschildi*	Misonne 1969		BMNH 501781	Extant
Muridae	Murinae	*Mallomys rothschildi*	Tate, 1936		AM104154	Extant
Muridae	Murinae	*Margaretamys beccarii*	Musser, 1981		AMNH 224061;	Extant
Muridae	Murinae	*Margaretamys beccarii*	Musser, 1981		AMNH 224064	Extant
Muridae	Murinae	*Margaretamys elegans*	Musser, 1981		AMNH 225143;	Extant
Muridae	Murinae	*Margaretamys parvus*	Musser, 1981		AMNH 226068;	Extant
Muridae	Murinae	*Mastacomys fuscus*	*		MNHN A2471	Extant
Muridae	Murinae	*Mastomys coucha*	*		MNHN 1971 509	Extant
Muridae	Murinae	*Maxomys inas*	*		MNHN 1977 206	Extant
Muridae	Murinae	*Maxomys wattsi*	Musser & Durden, 1991		MZB 12155	Extant
Muridae	Murinae	*Melasmothrix naso*	Musser, 1969		M12 4.4	Extant
Muridae	Murinae	*Melomys levipes*	*		MNHN 1985 1943	Extant
Muridae	Murinae	*Melomys lorentzii*	*		MNHN 1991 647	Extant
Muridae	Murinae	*Melomys rubex*	*		MNHN 1995 1560	Extant
Muridae	Murinae	*Melomys sp.*	Musser, 1982		length, 6.4	Extant
Muridae	Murinae	*Mesembriomys gouldi*	Missonne 1969		BMNH 8310192	Extant
Muridae	Murinae	*Micromys minutus*	*		MNHN 1932 4543	Extant
Muridae	Murinae	*Millardia meltada*	*		MNHN 1957 556	Extant

Collection number and references of the extant and extinct rodent used in this work. *indicates the specimens for which we take the photograph in the Museum collections (Musée National d'Histoire Naturelle, Paris; American Museum of Natural History, New York; National Museum of Natural History, Washington DC).

**Family**	**Subfamily**	***Species***	**References**	**Fossil site**	**Specimen ID**	**Extant/ Extinct**
Muridae	Murinae	*Muriculus imberbis*	*		1972 229	Extant
Muridae	Murinae	*Mus booduga*	*		MNHN 1968 5	Extant
Muridae	Murinae	*Mus bufo*	*		MNHN 1966 2215	Extant
Muridae	Murinae	*Mus caroli*	*		MNHN 1981 829	Extant
Muridae	Murinae	*Mus cervicolor*	*		MNHN 1981 839	Extant
Muridae	Murinae	*Mus confucianus*	*		MNHN 2007 218	Extant
Muridae	Murinae	*Mus cookii*	*		MNHN 1981 824	Extant
Muridae	Murinae	*Mus cypriacus*	*		MNHN 2005 834	Extant
Muridae	Murinae	*Mus dunni*	*		MNHN 1968 15	Extant
Muridae	Murinae	*Mus famulus*	*		MNHN 200 252	Extant
Muridae	Murinae	*Mus fragicauda*	*		MNHN 1999 1067	Extant
Muridae	Murinae	*Mus fulvidiventris*	*		MNHN 1973 321	Extant
Muridae	Murinae	*Mus goundae*	*		MNHN 1971 402	Extant
Muridae	Murinae	*Mus gratus*	*		MNHN 2005 686	Extant
Muridae	Murinae	*Mus haussa*	*		MNHN 1969 31	Extant
Muridae	Murinae	*Mus mahomet*	*		MNHN 1972 248	Extant
Muridae	Murinae	*Mus mattheyi*	*		MNHN 1972 732	Extant
Muridae	Murinae	*Mus minutoides*	*		MNHN 1970 185	Extant
Muridae	Murinae	*Mus musculoides*	*		MNHN 1977 471	Extant
Muridae	Murinae	*Mus musculus*	*		MNHN 1933 1871	Extant
Muridae	Murinae	*Mus musculus*	*		MNHN 1933 1893	Extant
Muridae	Murinae	*Mus musculus*	*		MNHN 1942 353	Extant

Collection number and references of the extant and extinct rodent used in this work. *indicates the specimens for which we take the photograph in the Museum collections (Musée National d'Histoire Naturelle, Paris; American Museum of Natural History, New York; National Museum of Natural History, Washington DC).

**Family**	**Subfamily**	***Species***	**References**	**Fossil site**	**Specimen ID**	**Extant/ Extinct**
Muridae	Murinae	*Mus musculus*	*		MNHN 1947 854	Extant
Muridae	Murinae	*Mus musculus*	*		MNHN 1947 855	Extant
Muridae	Murinae	*Mus musculus*	*		MNHN 1953 811	Extant
Muridae	Murinae	*Mus musculus*	*		MNHN 1953 812	Extant
Muridae	Murinae	*Mus musculus*	*		MNHN 1956 613	Extant
Muridae	Murinae	*Mus musculus*	*		MNHN 1957 1323	Extant
Muridae	Murinae	*Mus musculus*	*		MNHN 1957 369	Extant
Muridae	Murinae	*Mus musculus*	*		MNHN 1957 371	Extant
Muridae	Murinae	*Mus musculus*	*		MNHN 1957 373	Extant
Muridae	Murinae	*Mus musculus*	*		MNHN 1957 380	Extant
Muridae	Murinae	*Mus musculus*	*		MNHN 1957 545	Extant
Muridae	Murinae	*Mus musculus*	*		MNHN 1958 358	Extant
Muridae	Murinae	*Mus musculus*	*		MNHN 1958 359	Extant
Muridae	Murinae	*Mus musculus*	*		MNHN 1961 1064	Extant
Muridae	Murinae	*Mus musculus*	*		MNHN 1961 1065	Extant
Muridae	Murinae	*Mus musculus*	*		MNHN 1969 101	Extant
Muridae	Murinae	*Mus musculus*	*		MNHN 1969 102	Extant
Muridae	Murinae	*Mus musculus*	*		MNHN 1969 103	Extant
Muridae	Murinae	*Mus musculus*	*		MNHN 1970 334	Extant
Muridae	Murinae	*Mus musculus*	*		MNHN 1973 344	Extant
Muridae	Murinae	*Mus musculus*	*		MNHN 1980 207	Extant
Muridae	Murinae	*Mus musculus*	*		MNHN 1991 1185	Extant

Collection number and references of the extant and extinct rodent used in this work. *indicates the specimens for which we take the photograph in the Museum collections (Musée National d'Histoire Naturelle, Paris; American Museum of Natural History, New York; National Museum of Natural History, Washington DC).

**Family**	**Subfamily**	***Species***	**References**	**Fossil site**	**Specimen ID**	**Extant/ Extinct**
Muridae	Murinae	*Mus musculus*	*		MNHN 2000 253	Extant
Muridae	Murinae	*Mus Nannomys*	*		MNHN 1970 99	Extant
Muridae	Murinae	*Mus natalensis*	*		MNHN A7463	Extant
Muridae	Murinae	*Mus oubanguii*	*		MNHN 1971 446	Extant
Muridae	Murinae	*Mus praetextus*	*		MNHN 1991 1166	Extant
Muridae	Murinae	*Mus Pyromys*	*		MNHN 1997 2067	Extant
Muridae	Murinae	*Mus setulosus*	*		MNHN 1964 12	Extant
Muridae	Murinae	*Mus shortidgei*	*		MNHN 1981 825	Extant
Muridae	Murinae	*Mus spretus*	*		MNHN 1980 357	Extant
Muridae	Murinae	*Mus spretus*	*		MNHN 1980 417	Extant
Muridae	Murinae	*Mus tenellus*	*		MNHN 1977 27	Extant
Muridae	Murinae	*Mus triton*	*		MNHN 1996 414	Extant
Muridae	Murinae	*Mylomys dybowskii*	*		1993 2468	Extant
Muridae	Murinae	*Myomys daltoni*	*		1982 697	Extant
Muridae	Murinae	*Myomys daltoni*	*		1992 1573	Extant
Muridae	Murinae	*Myomys derooi*	*		1964 359	Extant
Muridae	Murinae	*Myomys yemenni*	*		1996 301	Extant
Muridae	Murinae	*Nesioka indica*	*		1958 80	Extant
Muridae	Murinae	*Nesoromys ceramicus*	Missonne 1969		BMNH	Extant
Muridae	Murinae	*Nivivemter coxingi*	*		MNHN 1874 634	Extant
Muridae	Murinae	*Niviventer cremoriventer*	Musser, 1981		AMNH 103579	Extant
Muridae	Murinae	*Niviventer fulvescens*	*		MNHN 1981 1292	Extant

Collection number and references of the extant and extinct rodent used in this work. *indicates the specimens for which we take the photograph in the Museum collections (Musée National d'Histoire Naturelle, Paris; American Museum of Natural History, New York; National Museum of Natural History, Washington DC).

**Family**	**Subfamily**	***Species***	**References**	**Fossil site**	**Specimen ID**	**Extant/ Extinct**
Muridae	Murinae	*Notomys alexis*	*		MNHN 2001 471	Extant
Muridae	Murinae	*Notomys mitchelli*	*		MNHN 1882 2172	Extant
Muridae	Murinae	*Oenomys hypoxanthus*	*		MNHN 1995 3002	Extant
Muridae	Murinae	*Papagonomys verhoeyeni*	Missone, 1969		Missonne	Extant
Muridae	Murinae	*Paraethomys meini*	Adrover, 1986		ALD 65	Extant
Muridae	Murinae	*Paraethomys meini*	Adrover, 1986		AR3 32	Extant
Muridae	Murinae	*Paraethomys meini*	Adrover, 1986		VAL 280	Extant
Muridae	Murinae	*Parahydromys asper*	Helgen, 2005			Extant
Muridae	Murinae	*Paulamys sp.*	Kitchener et al., 1991		WAMM32000	Extant
Muridae	Murinae	*Pelomys fallax*	*		MNHN 1952 881	Extant
Muridae	Murinae	*Phloeomys cumingi*	*		MNHN 1962 2564	Extant
Muridae	Murinae	*Phloeomys pallidus*	Missone, 1969		BMNH 97317	Extant
Muridae	Murinae	*Phloeomys sp.*	Musser & Headley 1992		AMNH242103	Extant
Muridae	Murinae	*Pitecheir melanurus*	*		MNHN 1894 1663	Extant
Muridae	Murinae	*Pithecheirops otion*	Emonds 1993			Extant
Muridae	Murinae	*Pogonomelomys bruijnii*	Helgen et al 2008		MSNG 3677	Extant
Muridae	Murinae	*Pogonomelomys LMNH*	Missonne 1969		LMNH 217	Extant
Muridae	Murinae	*Pogonomys fergussoniensis*	Missone, 1969		BMNH 501176	Extant
Muridae	Murinae	*Pogonomys forbesi*	Tate, 1936		79828	Extant
Muridae	Murinae	*Pogonomys lepidus*	Tate, 1936		AM104202	Extant
Muridae	Murinae	*Pogonomys macrourus*	*		MNHN 2005 823	Extant

Collection number and references of the extant and extinct rodent used in this work. *indicates the specimens for which we take the photograph in the Museum collections (Musée National d'Histoire Naturelle, Paris; American Museum of Natural History, New York; National Museum of Natural History, Washington DC).

**Family**	**Subfamily**	***Species***	**References**	**Fossil site**	**Specimen ID**	**Extant/ Extinct**
Muridae	Murinae	*Pogonomys sylvestris*	Tate, 1936		AM79757	Extant
Muridae	Murinae	*Praomys daltoni*	*		MNHN 1982 697	Extant
Muridae	Murinae	*Praomys daltoni*	*		MNHN 1992 1573	Extant
Muridae	Murinae	*Praomys derooi*	*		MNHN 1964 359	Extant
Muridae	Murinae	*Praomys tullbergi*	*		MNHN 1992 1438	Extant
Muridae	Murinae	*Praomys yemenni*	*		MNHN 1996 301	Extant
Muridae	Murinae	*Pseudohydromys patriciae*	Helgen & Helgen, 2009		AM M26991	Extant
Muridae	Murinae	*Pseudomys australis*	*		MNHN 1995 1561	Extant
Muridae	Murinae	*Rattus andersoni*	*		MNHN 1896 2098	Extant
Muridae	Murinae	*Rattus annandalei*	*		MNHN 1981 250	Extant
Muridae	Murinae	*Rattus argentiventer*	*		MNHN 1969 150	Extant
Muridae	Murinae	*Rattus confucianus*	*		MNHN 1929 464	Extant
Muridae	Murinae	*Rattus exiguus*	*		MNHN 1981 159	Extant
Muridae	Murinae	*Rattus exulans*	*		MNHN 1991 1191	Extant
Muridae	Murinae	*Rattus exulans*	*		MNHN 2009 117	Extant
Muridae	Murinae	*Rattus fuscipes*	*		MNHN 1846 1364	Extant
Muridae	Murinae	*Rattus koratensis*	*		MNHN 1990 540	Extant
Muridae	Murinae	*Rattus niobe*	Tate, 1936		AM104289	Extant
Muridae	Murinae	*Rattus nitidus*	*		MNHN 1899 122	Extant
Muridae	Murinae	*Rattus norvergicus*	*		MNHN 1862 472A	Extant
Muridae	Murinae	*Rattus norvergicus*	*		MNHN 1873 234	Extant

Collection number and references of the extant and extinct rodent used in this work. *indicates the specimens for which we take the photograph in the Museum collections (Musée National d'Histoire Naturelle, Paris; American Museum of Natural History, New York; National Museum of Natural History, Washington DC).

**Family**	**Subfamily**	***Species***	**References**	**Fossil site**	**Specimen ID**	**Extant/ Extinct**
Muridae	Murinae	*Rattus norvergicus*	*		MNHN 1874 633	Extant
Muridae	Murinae	*Rattus norvergicus*	*		MNHN 1903 35	Extant
Muridae	Murinae	*Rattus norvergicus*	*		MNHN 1933 2110	Extant
Muridae	Murinae	*Rattus norvergicus*	*		MNHN 1986 1204	Extant
Muridae	Murinae	*Rattus norvergicus*	*		MNHN 1995 3266	Extant
Muridae	Murinae	*Rattus rajah*	*		MNHN 1996 2292	Extant
Muridae	Murinae	*Rattus rattus*	*		MNHN 1902 1235	Extant
Muridae	Murinae	*Rattus rattus*	*		MNHN 1902 511	Extant
Muridae	Murinae	*Rattus rattus*	*		MNHN 1911 2352A	Extant
Muridae	Murinae	*Rattus rattus*	*		MNHN 1952 512	Extant
Muridae	Murinae	*Rattus rattus*	*		MNHN 1962 1841	Extant
Muridae	Murinae	*Rattus rattus*	*		MNHN 1994 840	Extant
Muridae	Murinae	*Rattus rattus*	*		MNHN 2007 1099	Extant
Muridae	Murinae	*Rattus rattus*	*		MNHN 2007 311	Extant
Muridae	Murinae	*Rattus sikkimensis*	*		MNHN 1995 2833	Extant
Muridae	Murinae	*Rattus sladeni*	*		MNHN 1997 79	Extant
Muridae	Murinae	*Rattus tanezumi*	*		MNHN 1874 616	Extant
Muridae	Murinae	*Rattus tiomanicus*	*		MNHN 1981 273	Extant
Muridae	Murinae	*Rattus turkestanicus*	*		MNHN 1991 1319	Extant
Muridae	Murinae	*Rattus verecundus*	Tate, 1936		AM104277	Extant
Muridae	Murinae	*Rhabdomys pumilo*	*		MNHN 2005 688	Extant
Muridae	Murinae	*Rhabdomys pumio*	*		MNHN 2000 837	Extant

Collection number and references of the extant and extinct rodent used in this work. *indicates the specimens for which we take the photograph in the Museum collections (Musée National d'Histoire Naturelle, Paris; American Museum of Natural History, New York; National Museum of Natural History, Washington DC).

**Family**	**Subfamily**	***Species***	**References**	**Fossil site**	**Specimen ID**	**Extant/ Extinct**
Muridae	Murinae	*Rhagamys orthodon*	Missonne 1969			Extant
Muridae	Murinae	*Rhynchomys sp.*	Musser & Headley 1992		FMNH 62289	Extant
Muridae	Murinae	*Saxatilomys paulinae*	Musser et al 2005		BMNH 2000.292	Extant
Muridae	Murinae	*Solomys sapientis*	Missone, 1969		IRSNB 6368	Extant
Muridae	Murinae	*Sommeromys macrorhinos*	Musser et al., 2002			Extant
Muridae	Murinae	*Spelaeomys florensis*	Missone, 1969			Extant
Muridae	Murinae	*Srilankamys ohiensis*	Musser 1981			Extant
Muridae	Murinae	*Stenocephalomys albicaudata*	*		1972 215	Extant
Muridae	Murinae	*Stochomys longicaudatus*	*		MNHN 1996 498	Extant
Muridae	Murinae	*Stochomys longicaudatus*	*		MNHN 1996 501	Extant
Muridae	Murinae	*Sundamys mulleri*	*		MNHN 1990 569	Extant
Muridae	Murinae	*Tarsomys 144617*	Musser & Headley 1992		144617	Extant
Muridae	Murinae	*Tarsomys 5959*	Musser & Headley 1992		5959	Extant
Muridae	Murinae	*Tateomys macrocercus*	Musser et al., 2002		AMNH 225077	Extant
Muridae	Murinae	*Thallomys paeduculus*	*		MNHN 1977 24	Extant
Muridae	Murinae	*Thammomys sp.*	*		MNHN AC1967 362	Extant
Muridae	Murinae	*Thamnomys rutilans*	*		1966 244	Extant
Muridae	Murinae	*Tokudaia sp.*	Kaneko, 2001			Extant

Collection number and references of the extant and extinct rodent used in this work. *indicates the specimens for which we take the photograph in the Museum collections (Musée National d'Histoire Naturelle, Paris; American Museum of Natural History, New York; National Museum of Natural History, Washington DC).

**Family**	**Subfamily**	***Species***	**References**	**Fossil site**	**Specimen ID**	**Extant/ Extinct**
Muridae	Murinae	*Tryphomys USNM536765*	Musser & Headley 1992		USNM 536765	Extant
Muridae	Murinae	*Uromys caudimaculatus*	Missone, 1969		IRSNB 4007	Extant
Muridae	Murinae	*Uromys validus*	Tate, 1936		AM104507	Extant
Muridae	Murinae	*Vandeluria oleracea*	*		MNHN 1969 276	Extant
Muridae	Murinae	*Vernaya fulva*	Missone, 1969		AMNH 115467	Extant
Muridae	Murinae	*Zelotomys hildegardeae*	*		MNHN 2000 114	Extant
Muridae	Murinae	*Zyzomys rackhami*	Godhelp, 1997			Extant
Muridae		*Anthracomys lorenzi*	Casanovas-Vilar et al., 2011	Fiume Santo	FS-779	Fossil
Muridae		*Anthracomys majori*	Casanovas-Vilar et al., 2011	Monte Bamboli	B5	Fossil
Muridae		*Castillomys cf. crusafonti*	García Alix, 2006; García Alix et al., 2008 lethaia	Barranco de Blas	BLS-6 10	Fossil
Muridae		*Castillomys cf. crusafonti*	Minwer-Barakat et al., 2005 JVP	Tollo Chiclana 1B	TCH-1B 356	Fossil
Muridae		*Castillomys crusafonti*	van der Weerd, 1970	Caravaca	1081	Fossil
Muridae		*Castillomys gracilis*	Martín-Suárez, 1988	Botardo-C	Bo-C 08	Fossil
Muridae		*Castillomys gracilis*	Martín-Suárez, 1988	Botardo-C	Bo-C 09	Fossil
Muridae		*Castillomys gracilis*	García-Alix, 2006	Calicasas 3	CLC-3 30	Fossil
Muridae		*Castillomys gracilis*	Bachelet, 1990	La Gloria 4	LG4 112	Fossil

Collection number and references of the extant and extinct rodent used in this work. *indicates the specimens for which we take the photograph in the Museum collections (Musée National d'Histoire Naturelle, Paris; American Museum of Natural History, New York; National Museum of Natural History, Washington DC).

**Family**	**Subfamily**	***Species***	**References**	**Fossil site**	**Specimen ID**	**Extant/ Extinct**
Muridae		*Castromys littoralis*	Freudenthal & Martin-Suarez,1999	Crevillente 17	RGM 413 09	Fossil
Muridae		*Castromys littoralis*	Martín-Suárez & Freudenthal, 1994	Crevillente 22	CR22-24	Fossil
Muridae		*Castromys littoralis*	García-Alix, 2006; García-Alix et al., 2008a	Jun 2B	JUN-2B 18	Fossil
Muridae		*Castromys littoralis*	García-Alix, 2006; García-Alix et al., 2008a	Jun 2B	JUN-2B 19	Fossil
Muridae		*Huerzelerimys minor*	Mein et al., 1993	Ambérieu 2C		Fossil
Muridae		*Huerzelerimys minor*	Mein et al., 1993	Ambérieu 2C		Fossil
Muridae		*Huerzelerimys minor*	Mein et al., 1993	Cascante		Fossil
Muridae		*Huerzelerimys minor*	Mein et al., 1993	Cortijo de Piedra 2		Fossil
Muridae		*Huerzelerimys minor*	Mein et al., 1993	Cucalón		Fossil
Muridae		*Huerzelerimys minor*	Adrover et al., 1993	La Gloria 4	LG4 15	Fossil
Muridae		*Huerzelerimys turolensis*	Adrover, 1986	Aljezar B	AB 921	Fossil
Muridae		*Huerzelerimys turolensis*	Adrover, 1986	Aljezar B	AB 932	Fossil
Muridae		*Huerzelerimys turolensis*	Adrover, 1986	Aljezar B	AB 933	Fossil
Muridae		*Huerzelerimys turolensis*	Adrover, 1986	Aljezar B	AB 969	Fossil
Muridae		*Huerzelerimys turolensis*	Adrover, 1986	Aljezar B	AB 970	Fossil
Muridae		*Huerzelerimys turolensis*	Adrover, 1986	Aljezar B	AB 974	Fossil

Collection number and references of the extant and extinct rodent used in this work. *indicates the specimens for which we take the photograph in the Museum collections (Musée National d'Histoire Naturelle, Paris; American Museum of Natural History, New York; National Museum of Natural History, Washington DC).

**Family**	**Subfamily**	***Species***	**References**	**Fossil site**	**Specimen ID**	**Extant/ Extinct**
Muridae		*Huerzelerimys turolensis*	Adrover, 1986	Aljezar B	AB 974	Fossil
Muridae		*Huerzelerimys turolensis*	Adrover, 1986	Aljezar B	AB944	Fossil
Muridae		*Huerzelerimys turolensis*	Adrover, 1986	Aljezar B	AB975	Fossil
Muridae		*Huerzelerimys turolensis*	Aguilar et al., 1995	Castelnou 1	CTN56	Fossil
Muridae		*Huerzelerimys turolensis*	Martín-Suárez & Freudenthal, 1993	Crevillente 15	RGM 403 833	Fossil
Muridae		*Huerzelerimys vireti*	Martín-Suárez & Freudenthal, 1993	Crevillente 2	RGM 402 373	Fossil
Muridae		*Huerzelerimys vireti*	Martín-Suárez & Freudenthal, 1993	Crevillente 2	RGM 402 384	Fossil
Muridae		*Huerzelerimys vireti*	Martín-Suárez & Freudenthal, 1993	Crevillente 4B	RGM 404 046	Fossil
Muridae		*Huerzelerimys vireti*	Adrover, 1986	Los Aguanaces	LA 300	Fossil
Muridae		*Huerzelerimys vireti*	Adrover, 1986	Los Aguanaces	LA 301	Fossil
Muridae		*Huerzelerimys vireti*	Adrover, 1986	Los Aguanaces	LA 306	Fossil
Muridae		*Huerzelerimys vireti*	Alcalá et al., 1990	Puente Minero	PM-1424	Fossil
Muridae		*Huerzelerimys vireti*	Adrover, 1986	Vivero de Pinos	VP 312	Fossil
Muridae		*Huerzelerimys vireti*	Adrover, 1986	Vivero de Pinos	VP 315	Fossil
Muridae		*Occitanomys adroveri*	Adrover, 1986	Aljezar B	AB 5	Fossil
Muridae		*Occitanomys adroveri*	Adrover, 1986	Aljezar B	AB 61	Fossil
Muridae		*Occitanomys adroveri*	Adrover, 1986	Aljezar B	AB 78	Fossil
Muridae		*Occitanomys adroveri*	Martín-Suárez, 1988	Botardo-C	Bo-C 16	Fossil

Collection number and references of the extant and extinct rodent used in this work. *indicates the specimens for which we take the photograph in the Museum collections (Musée National d'Histoire Naturelle, Paris; American Museum of Natural History, New York; National Museum of Natural History, Washington DC).

**Family**	**Subfamily**	***Species***	**References**	**Fossil site**	**Specimen ID**	**Extant/ Extinct**
Muridae		*Occitanomys adroveri*	Aguilar et al., 1991b	Castelnou 3	CTN3 155	Fossil
Muridae		*Occitanomys adroveri*	Martín-Suárez & Freudenthal, 1993	Crevillente 15	RGM 403 670	Fossil
Muridae		*Occitanomys adroveri*	Martín-Suárez & Freudenthal, 1993	Crevillente 15	RGM 403 679	Fossil
Muridae		*Occitanomys adroveri*	Freudenthal & Martín-Suárez, 1999	Crevillente 17	RGM 413 248	Fossil
Muridae		*Occitanomys adroveri*	Martín-Suárez & Freudenthal, 1993	Crevillente 17	RGM 413 345	Fossil
Muridae		*Occitanomys adroveri*	García-Alix, 2006	Otura-1	OTU-1 90	Fossil
Muridae		*Occitanomys adroveri*	García-Alix, 2006; García-Alix et al., 2008a	Otura-1	OTU-1 44	Fossil
Muridae		*Occitanomys alcalai*	Adrover et al., 1993	La Gloria 4	LG4 26	Fossil
Muridae		*Occitanomys alcalai*	Adrover et al., 1993	La Gloria 4	LG4 31	Fossil
Muridae		*Occitanomys alcalai*	García-Alix, 2006; García-Alix et al., 2008a	Purcal-4	PUR-4 39	Fossil
Muridae		*Occitanomys alcalai*	Minwer-Barakat et al., 2009	Rambla de Chimeneas 3	RCH-3 17	Fossil
Muridae		*Occitanomys faillati*	Aguilar et al., 1995	Castelnou 1	CTN1	Fossil

Collection number and references of the extant and extinct rodent used in this work. *indicates the specimens for which we take the photograph in the Museum collections (Musée National d'Histoire Naturelle, Paris; American Museum of Natural History, New York; National Museum of Natural History, Washington DC).

Family	Subfamily	*Species*	References	Fossil site	Specimen ID	Extant/ Extinct
Muridae		*Occitanomys sondaari*	Martín-Suárez & Freudenthal, 1993	Crevillente 2	RGM 402 596	Fossil
Muridae		*Occitanomys sondaari*	Martín-Suárez & Freudenthal, 1993	Crevillente 2	RGM 402 598	Fossil
Muridae		*Occitanomys sondaari*	Martín-Suárez & Freudenthal, 1993	Crevillente 2	RGM 402 616	Fossil
Muridae		*Occitanomys sondaari*	Martín-Suárez & Freudenthal, 1993	Crevillente 4B	RGM 404 459	Fossil
Muridae		*Occitanomys sondaari*	Martín-Suárez & Freudenthal, 1993	Crevillente 4B	RGM 404 466	Fossil
Muridae		*Occitanomys sondaari*	Adrover, 1986	Los Aguanaces	LA 1	Fossil
Muridae		*Occitanomys sondaari*	Adrover, 1986	Los Aguanaces	LA 22	Fossil
Muridae		*Occitanomys sondaari*	Adrover, 1986	Los Aguanaces	LA 243	Fossil
Muridae		*Occitanomys sondaari*	Adrover, 1986	Los Aguanaces	LA 245	Fossil
Muridae		*Occitanomys sondaari*	Adrover, 1986	Los Aguanaces	LA 246	Fossil
Muridae		*Occitanomys sondaari*	Adrover, 1986	Los Aguanaces	LA 50	Fossil
Muridae		*Occitanomys sondaari*	Alcalá et al., 1990	Puente Minero	PM-1160	Fossil
Muridae		*Occitanomys sondaari*	Alcalá et al., 1990	Puente Minero	PM-1173	Fossil
Muridae		*Occitanomys sondaari*	Alcalá et al., 1990	Puente Minero	PM-1178	Fossil
Muridae		*Occitanomys sondaari*	Adrover, 1986	Vivero de Pinos	VP 10	Fossil
Muridae		*Occitanomys sondaari*	Adrover, 1986	Vivero de Pinos	VP 262	Fossil
Muridae		*Occitanomys sondaari*	Adrover, 1986	Vivero de Pinos	VP 274	Fossil

Collection number and references of the extant and extinct rodent used in this work. *indicates the specimens for which we take the photograph in the Museum collections (Musée National d'Histoire Naturelle, Paris; American Museum of Natural History, New York; National Museum of Natural History, Washington DC).

Family	Subfamily	*Species*	References	Fossil site	Specimen ID	Extant/ Extinct
Muridae		*Occitanomys sondaari*	Adrover, 1986	Vivero de Pinos	VP 275	Fossil
Muridae		*Occitanomys sondaari*	Adrover, 1986	Vivero de Pinos	VP 30	Fossil
Muridae		*Occitanomys sondaari*	Adrover, 1986	Vivero de Pinos	VP 43	Fossil
Muridae		*Paraethomis cf. meini*	Ruíz Bustos et al., 1984	Gorafe A	G21	Fossil
Muridae		*Paraethomys aff. abaigari*	García-Alix, 2006; García-Alix et al., 2008a	Purcal-13	PUR-13 41	Fossil
Muridae		*Paraethomys meini*	Martín-Suárez, 1988	Botardo-C	Bo-C 23	Fossil
Muridae		*Paraethomys meini*	Adrover et al., 1993	La Gloria 4	LG4 54	Fossil
Muridae		*Paraethomys meini*	García-Alix, 2006	Mina 4	MNA-4 61	Fossil
Muridae		*Paraethomys meini*	García-Alix, 2006; García-Alix et al., 2008a	Purcal-3	PUR-3 25	Fossil
Muridae		*Progonomys cathalai*	Wessels, 2009	Altintas 1		Fossil
Muridae		*Progonomys cathalai*	Wessels, 2009	Altintas 1		Fossil
Muridae		*Progonomys cathalai*	Wessels, 2009	Altintas 1		Fossil
Muridae		*Progonomys cathalai*	Wessels, 2009	Altintas 1		Fossil
Muridae		*Progonomys cathalai*	Wessels, 2009	Altintas 1		Fossil
Muridae		*Progonomys cathalai*	Wessels, 2009	Altintas 1		Fossil
Muridae		*Progonomys cathalai*	Wessels, 2009	Altintas 1		Fossil
Muridae		*Progonomys cathalai*	Wessels, 2009	Altintas 2		Fossil

Collection number and references of the extant and extinct rodent used in this work. *indicates the specimens for which we take the photograph in the Museum collections (Musée National d'Histoire Naturelle, Paris; American Museum of Natural History, New York; National Museum of Natural History, Washington DC).

Family	Subfamily	*Species*	References	Fossil site	Specimen ID	Extant/ Extinct
Muridae		*Progonomys cathalai*	Wessels, 2009	Altintas 2		Fossil
Muridae		*Progonomys cathalai*	Mein et al., 1993	Ambérieu		Fossil
Muridae		*Progonomys cathalai*	Mein et al., 1993	Bayraktepe	Bayraktepe II	Fossil
Muridae		*Progonomys cathalai*	Mein et al., 1993	Biodrak		Fossil
Muridae		*Progonomys cathalai*	Wessels, 2009	Kütahya		Fossil
Muridae		*Progonomys cathalai*	Wessels, 2009	Kütahya		Fossil
Muridae		*Progonomys cathalai*	Mein et al., 1993	Masia del barbo 2B		Fossil
Muridae		*Progonomys cathalai*	Mein et al., 1993	Montredon		Fossil
Muridae		*Progonomys cathalai*	Mein et al., 1993	Soblay		Fossil
Muridae		*Progonomys cathalai*	Agustí, 1981	Torrent de Febulines		Fossil
Muridae		*Progonomys cf. woelferi*	Guerra-Merchán et al., 2001	Racor	Ra-4	Fossil
Muridae		*Progonomys clauzoni*	Lazzari et al., 2010	Lo Fournas 16M	FOU16M 758	Fossil
Muridae		*Progonomys clauzoni*	Lazzari et al., 2010	Lo Fournas 16M	FOU16M 759	Fossil
Muridae		*Progonomys clauzoni*	Lazzari et al., 2010	Lo Fournas 16M	FOU16M 760	Fossil
Muridae		*Progonomys clauzoni*	Lazzari et al., 2010	Lo Fournas 16M	FOU16M 761	Fossil
Muridae		*Progonomys clauzoni*	Lazzari et al., 2010	Lo Fournas 16M	FOU16M 762	Fossil
Muridae		*Progonomys clauzoni*	Lazzari et al., 2010	Lo Fournas 16M	FOU16M 763	Fossil
Muridae		*Progonomys clauzoni*	Lazzari et al., 2010	Lo Fournas 16M	FOU16M 764	Fossil
Muridae		*Progonomys clauzoni*	Lazzari et al., 2010	Lo Fournas 16M	FOU16M 769	Fossil
Muridae		*Progonomys clauzoni*	Lazzari et al., 2010	Lo Fournas 16M	FOU16M 772	Fossil
Muridae		*Progonomys clauzoni*	Lazzari et al., 2010	Lo Fournas 16M	FOU16M 778	Fossil

Collection number and references of the extant and extinct rodent used in this work. *indicates the specimens for which we take the photograph in the Museum collections (Musée National d'Histoire Naturelle, Paris; American Museum of Natural History, New York; National Museum of Natural History, Washington DC).

**Family**	**Subfamily**	***Species***	**References**	**Fossil site**	**Specimen ID**	**Extant/ Extinct**
Muridae		*Progonomys clauzoni*	Lazzari et al., 2010	Lo Fournas 16M	FOU16M 780	Fossil
Muridae		*Progonomys clauzoni*	Lazzari et al., 2010	Lo Fournas 16M	FOU16M 790	Fossil
Muridae		*Progonomys clauzoni*	Lazzari et al., 2010	Lo Fournas 16M	FOU16M 795	Fossil
Muridae		*Progonomys clauzoni*	Lazzari et al., 2010	Lo Fournas 16M	FOU16M 820	Fossil
Muridae		*Progonomys clauzoni*	Lazzari et al., 2010	Lo Fournas 16M	FOU16M 846	Fossil
Muridae		*Progonomys clauzoni*	Lazzari et al., 2010	Lo Fournas 6a	FOU6a 103	Fossil
Muridae		*Progonomys clauzoni*	Lazzari et al., 2010	Lo Fournas 6a	FOU6a 114	Fossil
Muridae		*Progonomys clauzoni*	Lazzari et al., 2010	Lo Fournas 6a	FOU6a 155	Fossil
Muridae		*Progonomys clauzoni*	Lazzari et al., 2010	Lo Fournas 6a	FOU6a 156	Fossil
Muridae		*Progonomys clauzoni*	Lazzari et al., 2010	Lo Fournas 6a	FOU6a 157	Fossil
Muridae		*Progonomys clauzoni*	Lazzari et al., 2010	Lo Fournas 6a	FOU6a 158	Fossil
Muridae		*Progonomys clauzoni*	Lazzari et al., 2010	Lo Fournas 6a	FOU6a 159	Fossil
Muridae		*Progonomys clauzoni*	Lazzari et al., 2010	Lo Fournas 6a	FOU6a 160	Fossil
Muridae		*Progonomys clauzoni*	Lazzari et al., 2010	Lo Fournas 6b	FOU6b 16	Fossil
Muridae		*Progonomys clauzoni*	Lazzari et al., 2010	Lo Fournas 6b	FOU6b 19	Fossil
Muridae		*Progonomys clauzoni*	Lazzari et al., 2010	Lo Fournas 6b	FOU6b 28	Fossil
Muridae		*Progonomys clauzoni*	Lazzari et al., 2010	Lo Fournas 6b	FOU6b 44	Fossil
Muridae		*Progonomys clauzoni*	Lazzari et al., 2010	Lo Fournas 6b	FOU6b 45	Fossil
Muridae		*Progonomys clauzoni*	Lazzari et al., 2010	Lo Fournas 6b	FOU6b 46	Fossil
Muridae		*Progonomys clauzoni*	Lazzari et al., 2010	Lo Fournas 6b	FOU6b 8	Fossil
Muridae		*Progonomys clauzoni*	Lazzari et al., 2010	Lo Fournas 6c	FOU6c 1081	Fossil
Muridae		*Progonomys clauzoni*	Lazzari et al., 2010	Lo Fournas 6c	FOU6c 1082	Fossil

Collection number and references of the extant and extinct rodent used in this work. *indicates the specimens for which we take the photograph in the Museum collections (Musée National d'Histoire Naturelle, Paris; American Museum of Natural History, New York; National Museum of Natural History, Washington DC).

**Family**	**Subfamily**	***Species***	**References**	**Fossil site**	**Specimen ID**	**Extant/ Extinct**
Muridae		*Progonomys clauzoni*	Lazzari et al., 2010	Lo Fournas 6c	FOU6c 1083	Fossil
Muridae		*Progonomys clauzoni*	Lazzari et al., 2010	Lo Fournas 6c	FOU6c 160	Fossil
Muridae		*Progonomys clauzoni*	Lazzari et al., 2010	Lo Fournas 6c	FOU6c 196	Fossil
Muridae		*Progonomys clauzoni*	Lazzari et al., 2010	Lo Fournas 6c	FOU6c 206	Fossil
Muridae		*Progonomys clauzoni*	Lazzari et al., 2010	Lo Fournas 6c	FOU6c 207	Fossil
Muridae		*Progonomys clauzoni*	Lazzari et al., 2010	Lo Fournas 6c	FOU6c 210	Fossil
Muridae		*Progonomys hispanicus*	Antunes et al., 1992	Asseiceira		Fossil
Muridae		*Progonomys hispanicus*	Sesé, 2003	Belmonte	BE-3	Fossil
Muridae		*Progonomys hispanicus*	Sesé, 2003	Belmonte	BE-4	Fossil
Muridae		*Progonomys woelferi*	Mein et al., 1993	Kastellios		Fossil
Muridae		*Progonomys woelferi*	Mein et al., 1993	Kohfidisch		Fossil
Muridae		*Progonomys woelferi*	Mein et al., 1993	Torrent de Febulines		Fossil
Muridae		*Progonomys woelferi*	Mein et al., 1993	YGSP	YGSP 182A	Fossil
Muridae		*Rhagapodemus primaevus*	Aguilar et al., 1991b	Castenou 3	CTN 3 208	Fossil
Muridae		*Rhagapodemus sp.*	Adrover et al., 1993	La Gloria 4	LG4 121	Fossil
Muridae		*Stephanomys cf. cordii*	Ruíz Bustos et al., 1984	Gorafe A	G1	Fossil
Muridae		*Stephanomys dubari*	Aguilar et al., 1991b	Castenou 3	CTN 3 79	Fossil
Muridae		*Stephanomys dubari*	Aguilar et al., 1991b	Castenou 3	CTN 3 83	Fossil
Muridae		*Stephanomys dubari*	Aguilar et al., 1991b	Castenou 3	CTN 3 85	Fossil
Muridae		*Stephanomys dubari*	Aguilar et al., 1991b	Castenou 3	CTN 3 94	Fossil
Muridae		*Stephanomys dubari*	García-Alix, 2006	Dehesa-16	DHS-16 129	Fossil

Collection number and references of the extant and extinct rodent used in this work. *indicates the specimens for which we take the photograph in the Museum collections (Musée National d'Histoire Naturelle, Paris; American Museum of Natural History, New York; National Museum of Natural History, Washington DC).

**Family**	**Subfamily**	***Species***	**References**	**Fossil site**	**Specimen ID**	**Extant/ Extinct**
Muridae		*Stephanomys dubari*	García-Alix, 2006	Purcal-13	PUR-13 112	Fossil
Muridae		*Stephanomys ramblensis*	Sanz et al., 1992	Canteras de Iberia		Fossil
Muridae		*Stephanomys ramblensis*	Sanz et al., 1992	Canteras de Iberia		Fossil
Muridae		*Stephanomys ramblensis*	Adrover et al., 1993	La Gloria 5	LG5 103	Fossil
Muridae		*Stephanomys ramblensis*	García-Alix, 2006	Purcal-24	PUR-24 81	Fossil
Muridae		*Stephanomys stadii*	Mein & Michaux, 1979	Cucuron	FSL 65626	Fossil
Muridae		*Stephanomys stadii*	Mein & Michaux, 1979	Cucuron	FSL 65627	Fossil
Muridae		*Stephanomys stadii*	Mein & Michaux, 1979	Cucuron	FSL 65628	Fossil
Cricetidae	Cricetinae	*Allocricetulus curtatus*			33957	Extant
Cricetidae	Cricetinae	*Cricetus cricetus*			176483	Extant
Cricetidae	Cricetinae	*Mesocricetus auratus*			144771	Extant
Cricetidae	Cricetinae	*Phodopus campbelli*			84015	Extant
Cricetidae	Cricetinae	*Phodopus roborovskii*			155024	Extant
Cricetidae	Cricetinae	*Tscherskia triton*			56717	Extant
Cricetidae	Cricetinae	*Tscherskia triton*			219219	Extant
Cricetidae	Cricetinae	*Tscherskia triton*			252745	Extant
Cricetidae	Lophiomyinae	*Lophiomys imhausi*			33332	Extant
Cricetidae	Neotominae	*Baiomys musculus*			123475	Extant
Cricetidae	Neotominae	*Habromys lepturus*			182098	Extant

Collection number and references of the extant and extinct rodent used in this work. *indicates the specimens for which we take the photograph in the Museum collections (Musée National d'Histoire Naturelle, Paris; American Museum of Natural History, New York; National Museum of Natural History, Washington DC).

**Family**	**Subfamily**	***Species***	**References**	**Fossil site**	**Specimen ID**	**Extant/ Extinct**
Cricetidae	Neotominae	*Hodomys alleni*			171930	Extant
Cricetidae	Neotominae	*Isrhmomys pirrensis*			38032	Extant
Cricetidae	Neotominae	*Megadontomys thomasi*			254779	Extant
Cricetidae	Neotominae	*Nelsonia goldmani*			125813	Extant
Cricetidae	Neotominae	*Nelsonia sp.*			13096	Extant
Cricetidae	Neotominae	*Neotoma albigula*			7102	Extant
Cricetidae	Neotominae	*Neotomodon alstoni*			146938	Extant
Cricetidae	Neotominae	*Ochrotomys nuttalli*			5597	Extant
Cricetidae	Neotominae	*Onychomys leucogaster*			204631	Extant
Cricetidae	Neotominae	*Osdogonomys banderanus*			254722	Extant
Cricetidae	Neotominae	*Osdogonomys sp.*			172074	Extant
Cricetidae	Neotominae	*Peromyscus attwateri*			136510	Extant
Cricetidae	Neotominae	*Peromyscus boylii*			14547	Extant
Cricetidae	Neotominae	*Peromyscus boylii*			173834	Extant
Cricetidae	Neotominae	*Peromyscus boylii*			180545	Extant
Cricetidae	Neotominae	*Peromyscus californicus*			11601	Extant
Cricetidae	Neotominae	*Peromyscus californicus*			11602	Extant
Cricetidae	Neotominae	*Peromyscus californicus*			11603	Extant
Cricetidae	Neotominae	*Peromyscus californicus*			11604	Extant
Cricetidae	Neotominae	*Peromyscus californicus*			11605	Extant
Cricetidae	Neotominae	*Peromyscus californicus*			11606	Extant
Cricetidae	Neotominae	*Peromyscus californicus*			11607	Extant

Collection number and references of the extant and extinct rodent used in this work. *indicates the specimens for which we take the photograph in the Museum collections (Musée National d'Histoire Naturelle, Paris; American Museum of Natural History, New York; National Museum of Natural History, Washington DC).

Family	Subfamily	*Species*	References	Fossil site	Specimen ID	Extant/ Extinct
Cricetidae	Neotominae	*Peromyscus californicus*			11608	Extant
Cricetidae	Neotominae	*Peromyscus californicus*			11609	Extant
Cricetidae	Neotominae	*Peromyscus californicus*			11610	Extant
Cricetidae	Neotominae	*Peromyscus californicus*			11611	Extant
Cricetidae	Neotominae	*Peromyscus californicus*			11624	Extant
Cricetidae	Neotominae	*Peromyscus californicus*			139425	Extant
Cricetidae	Neotominae	*Peromyscus californicus*			139462	Extant
Cricetidae	Neotominae	*Peromyscus californicus*			2660	Extant
Cricetidae	Neotominae	*Peromyscus californicus*			2662	Extant
Cricetidae	Neotominae	*Peromyscus californicus*			2663	Extant
Cricetidae	Neotominae	*Peromyscus californicus*			28773	Extant
Cricetidae	Neotominae	*Peromyscus caniceps*			180645	Extant
Cricetidae	Neotominae	*Peromyscus crinitus*			275805	Extant
Cricetidae	Neotominae	*Peromyscus dickeyi*			180695	Extant
Cricetidae	Neotominae	*Peromyscus difficilis*			146888	Extant
Cricetidae	Neotominae	*Peromyscus eremicus*			180718	Extant
Cricetidae	Neotominae	*Peromyscus eremicus*			132305	Extant
Cricetidae	Neotominae	*Peromyscus eremicus*			132396	Extant
Cricetidae	Neotominae	*Peromyscus eremicus*			132397	Extant
Cricetidae	Neotominae	*Peromyscus eremicus*			132417	Extant
Cricetidae	Neotominae	*Peromyscus eremicus*			132423	Extant
Cricetidae	Neotominae	*Peromyscus eremicus*			132424	Extant

Collection number and references of the extant and extinct rodent used in this work. *indicates the specimens for which we take the photograph in the Museum collections (Musée National d'Histoire Naturelle, Paris; American Museum of Natural History, New York; National Museum of Natural History, Washington DC).

**Family**	**Subfamily**	***Species***	**References**	**Fossil site**	**Specimen ID**	**Extant/ Extinct**
Cricetidae	Neotominae	*Peromyscus eremicus*			132427	Extant
Cricetidae	Neotominae	*Peromyscus eremicus*			169582	Extant
Cricetidae	Neotominae	*Peromyscus eremicus*			169585	Extant
Cricetidae	Neotominae	*Peromyscus eremicus*			169600	Extant
Cricetidae	Neotominae	*Peromyscus eremicus*			173869	Extant
Cricetidae	Neotominae	*Peromyscus eremicus*			173894	Extant
Cricetidae	Neotominae	*Peromyscus eremicus*			175060	Extant
Cricetidae	Neotominae	*Peromyscus eremicus*			175061	Extant
Cricetidae	Neotominae	*Peromyscus eremicus*			175062	Extant
Cricetidae	Neotominae	*Peromyscus eremicus*			175504	Extant
Cricetidae	Neotominae	*Peromyscus eremicus*			175505	Extant
Cricetidae	Neotominae	*Peromyscus eremicus*			238352	Extant
Cricetidae	Neotominae	*Peromyscus eremicus*			238353	Extant
Cricetidae	Neotominae	*Peromyscus eremicus*			263391	Extant
Cricetidae	Neotominae	*Peromyscus eremicus*			263392	Extant
Cricetidae	Neotominae	*Peromyscus eremicus*			275916	Extant
Cricetidae	Neotominae	*Peromyscus eremicus*			2993	Extant
Cricetidae	Neotominae	*Peromyscus eremicus*			31872	Extant
Cricetidae	Neotominae	*Peromyscus eremicus*			31874	Extant
Cricetidae	Neotominae	*Peromyscus eremicus*			31875	Extant
Cricetidae	Neotominae	*Peromyscus eremicus*			31876	Extant
Cricetidae	Neotominae	*Peromyscus eremicus*			31877	Extant

Collection number and references of the extant and extinct rodent used in this work. *indicates the specimens for which we take the photograph in the Museum collections (Musée National d'Histoire Naturelle, Paris; American Museum of Natural History, New York; National Museum of Natural History, Washington DC).

**Family**	**Subfamily**	***Species***	**References**	**Fossil site**	**Specimen ID**	**Extant/ Extinct**
Cricetidae	Neotominae	*Peromyscus eremicus*			31879	Extant
Cricetidae	Neotominae	*Peromyscus eremicus*			31880	Extant
Cricetidae	Neotominae	*Peromyscus eremicus*			31881	Extant
Cricetidae	Neotominae	*Peromyscus eremicus*			31887	Extant
Cricetidae	Neotominae	*Peromyscus eremicus*			31916	Extant
Cricetidae	Neotominae	*Peromyscus eremicus*			31953	Extant
Cricetidae	Neotominae	*Peromyscus eremicus*			40476	Extant
Cricetidae	Neotominae	*Peromyscus eremicus*			40477	Extant
Cricetidae	Neotominae	*Peromyscus eremicus*			40478	Extant
Cricetidae	Neotominae	*Peromyscus eremicus*			40479	Extant
Cricetidae	Neotominae	*Peromyscus eremicus*			40482	Extant
Cricetidae	Neotominae	*Peromyscus eremicus*			4518	Extant
Cricetidae	Neotominae	*Peromyscus eremicus*			4519	Extant
Cricetidae	Neotominae	*Peromyscus eremicus*			4521	Extant
Cricetidae	Neotominae	*Peromyscus eremicus*			4532	Extant
Cricetidae	Neotominae	*Peromyscus eremicus*			4540	Extant
Cricetidae	Neotominae	*Peromyscus eremicus*			4543	Extant
Cricetidae	Neotominae	*Peromyscus eremicus*			4978	Extant
Cricetidae	Neotominae	*Peromyscus eremicus*			6439	Extant
Cricetidae	Neotominae	*Peromyscus eremicus*			6483	Extant
Cricetidae	Neotominae	*Peromyscus eremicus*			6484	Extant
Cricetidae	Neotominae	*Peromyscus eremicus*			6501	Extant

Collection number and references of the extant and extinct rodent used in this work. *indicates the specimens for which we take the photograph in the Museum collections (Musée National d'Histoire Naturelle, Paris; American Museum of Natural History, New York; National Museum of Natural History, Washington DC).

Family	Subfamily	*Species*	References	Fossil site	Specimen ID	Extant/ Extinct
Cricetidae	Neotominae	*Peromyscus eremicus*			68666	Extant
Cricetidae	Neotominae	*Peromyscus eremicus*			68667	Extant
Cricetidae	Neotominae	*Peromyscus eremicus*			68668	Extant
Cricetidae	Neotominae	*Peromyscus eremicus*			68669	Extant
Cricetidae	Neotominae	*Peromyscus eremicus*			94251	Extant
Cricetidae	Neotominae	*Peromyscus eremicus*			94252	Extant
Cricetidae	Neotominae	*Peromyscus eremicus*			94254	Extant
Cricetidae	Neotominae	*Peromyscus eremicus*			94255	Extant
Cricetidae	Neotominae	*Peromyscus eremicus*			94256	Extant
Cricetidae	Neotominae	*Peromyscus eremicus*			94257	Extant
Cricetidae	Neotominae	*Peromyscus eremicus*			94258	Extant
Cricetidae	Neotominae	*Peromyscus eremicus*			94259	Extant
Cricetidae	Neotominae	*Peromyscus eremicus*			94261	Extant
Cricetidae	Neotominae	*Peromyscus eremicus*			94262	Extant
Cricetidae	Neotominae	*Peromyscus eremicus*			94263	Extant
Cricetidae	Neotominae	*Peromyscus eremicus*			94264	Extant
Cricetidae	Neotominae	*Peromyscus eremicus*			94265	Extant
Cricetidae	Neotominae	*Peromyscus eremicus*			94266	Extant
Cricetidae	Neotominae	*Peromyscus eremicus*			94267	Extant
Cricetidae	Neotominae	*Peromyscus eremicus*			94268	Extant
Cricetidae	Neotominae	*Peromyscus eremicus*			94269	Extant
Cricetidae	Neotominae	*Peromyscus eremicus*			94270	Extant

Collection number and references of the extant and extinct rodent used in this work. *indicates the specimens for which we take the photograph in the Museum collections (Musée National d'Histoire Naturelle, Paris; American Museum of Natural History, New York; National Museum of Natural History, Washington DC).

Family	Subfamily	*Species*	References	Fossil site	Specimen ID	Extant/ Extinct
Cricetidae	Neotominae	*Peromyscus gossypinus*			145135	Extant
Cricetidae	Neotominae	*Peromyscus grandis*			79342	Extant
Cricetidae	Neotominae	*Peromyscus guardia*			180783	Extant
Cricetidae	Neotominae	*Peromyscus guatemalensis*			79198	Extant
Cricetidae	Neotominae	*Peromyscus leucopus*			17171	Extant
Cricetidae	Neotominae	*Peromyscus maniculatus*			125271	Extant
Cricetidae	Neotominae	*Peromyscus maniculatus*			166803	Extant
Cricetidae	Neotominae	*Peromyscus maniculatus*			166804	Extant
Cricetidae	Neotominae	*Peromyscus maniculatus*			166806	Extant
Cricetidae	Neotominae	*Peromyscus maniculatus*			166807	Extant
Cricetidae	Neotominae	*Peromyscus maniculatus*			166808	Extant
Cricetidae	Neotominae	*Peromyscus maniculatus*			166809	Extant
Cricetidae	Neotominae	*Peromyscus megalops*			146031	Extant
Cricetidae	Neotominae	*Peromyscus melanocarpus*			182105	Extant
Cricetidae	Neotominae	*Peromyscus melanotis*			146867	Extant
Cricetidae	Neotominae	*Peromyscus melanotis*			174865	Extant
Cricetidae	Neotominae	*Peromyscus mexicanus*			68550	Extant
Cricetidae	Neotominae	*Peromyscus ochraventer*			254761	Extant
Cricetidae	Neotominae	*Peromyscus pectoralis*			148671	Extant
Cricetidae	Neotominae	*Peromyscus perfulvus*			254768	Extant
Cricetidae	Neotominae	*Peromyscus polionotus*			250490	Extant
Cricetidae	Neotominae	*Peromyscus polius*			15864	Extant

Collection number and references of the extant and extinct rodent used in this work. *indicates the specimens for which we take the photograph in the Museum collections (Musée National d'Histoire Naturelle, Paris; American Museum of Natural History, New York; National Museum of Natural History, Washington DC).

**Family**	**Subfamily**	***Species***	**References**	**Fossil site**	**Specimen ID**	**Extant/ Extinct**
Cricetidae	Neotominae	*Peromyscus sejugis*			180646	Extant
Cricetidae	Neotominae	*Peromyscus spicilegus*			25458	Extant
Cricetidae	Neotominae	*Peromyscus stephani*			31960	Extant
Cricetidae	Neotominae	*Peromyscus stirtoni*			126346	Extant
Cricetidae	Neotominae	*Peromyscus truei*			11643	Extant
Cricetidae	Neotominae	*Peromyscus yucatanicus*			100351	Extant
Cricetidae	Neotominae	*Peromyscus yucatanicus*			100352	Extant
Cricetidae	Neotominae	*Peromyscus yucatanicus*			108006	Extant
Cricetidae	Neotominae	*Peromyscus yucatanicus*			108007	Extant
Cricetidae	Neotominae	*Peromyscus yucatanicus*			108008	Extant
Cricetidae	Neotominae	*Peromyscus yucatanicus*			108009	Extant
Cricetidae	Neotominae	*Peromyscus yucatanicus*			108010	Extant
Cricetidae	Neotominae	*Podomys floridanus*			258887	Extant
Cricetidae	Neotominae	*Podomys floridanus*			14316	Extant
Cricetidae	Neotominae	*Reithrodontomys sp.*			136995	Extant
Cricetidae	Neotominae	*Scotinomys teguina*			18134	Extant
Cricetidae	Neotominae	*Xenomys nelsoni*			45285	Extant
Cricetidae	Sigmodontinae	*Abrawayaomys ruschii*			552416	Extant
Cricetidae	Sigmodontinae	*Abrothrix andinus*			541611	Extant
Cricetidae	Sigmodontinae	*Abrothrix longipilis*			97707	Extant
Cricetidae	Sigmodontinae	*Abrothrix longipilis*			14296	Extant
Cricetidae	Sigmodontinae	*Aepeomys lugens*			22151	Extant

Collection number and references of the extant and extinct rodent used in this work. *indicates the specimens for which we take the photograph in the Museum collections (Musée National d'Histoire Naturelle, Paris; American Museum of Natural History, New York; National Museum of Natural History, Washington DC).

**Family**	**Subfamily**	***Species***	**References**	**Fossil site**	**Specimen ID**	**Extant/ Extinct**
Cricetidae	Sigmodontinae	*Aepeomys lugens*			387958	Extant
Cricetidae	Sigmodontinae	*Aepeomys lugens*			387961	Extant
Cricetidae	Sigmodontinae	*Akodon aerosus*			148341	Extant
Cricetidae	Sigmodontinae	*Akodon aerosus*			149006	Extant
Cricetidae	Sigmodontinae	*Akodon aerosus*			513596	Extant
Cricetidae	Sigmodontinae	*Akodon aerosus*			513597	Extant
Cricetidae	Sigmodontinae	*Akodon aerosus*			72890	Extant
Cricetidae	Sigmodontinae	*Akodon affinis*			199580	Extant
Cricetidae	Sigmodontinae	*Akodon affinis*			240043	Extant
Cricetidae	Sigmodontinae	*Akodon albiventer*			541628	Extant
Cricetidae	Sigmodontinae	*Akodon albiventer*			541634	Extant
Cricetidae	Sigmodontinae	*Akodon azarae*			236273	Extant
Cricetidae	Sigmodontinae	*Akodon azarae*			236300	Extant
Cricetidae	Sigmodontinae	*Akodon bogotensis*			280775	Extant
Cricetidae	Sigmodontinae	*Akodon bogotensis*			280792	Extant
Cricetidae	Sigmodontinae	*Akodon boliviensis*			268764	Extant
Cricetidae	Sigmodontinae	*Akodon boliviensis*			271407	Extant
Cricetidae	Sigmodontinae	*Akodon budini*			259612	Extant
Cricetidae	Sigmodontinae	*Akodon cursor*			531217	Extant
Cricetidae	Sigmodontinae	*Akodon dayi*			290926	Extant
Cricetidae	Sigmodontinae	*Akodon dayi*			390141	Extant
Cricetidae	Sigmodontinae	*Akodon fumeus*			290907	Extant

Collection number and references of the extant and extinct rodent used in this work. *indicates the specimens for which we take the photograph in the Museum collections (Musée National d’Histoire Naturelle, Paris; American Museum of Natural History, New York; National Museum of Natural History, Washington DC).

**Family**	**Subfamily**	***Species***	**References**	**Fossil site**	**Specimen ID**	**Extant/ Extinct**
Cricetidae	Sigmodontinae	*Akodon fumeus*			290927	Extant
Cricetidae	Sigmodontinae	*Akodon iniscatus*			236314	Extant
Cricetidae	Sigmodontinae	*Akodon kofordi*			172966	Extant
Cricetidae	Sigmodontinae	*Akodon lutescens*			259623	Extant
Cricetidae	Sigmodontinae	*Akodon mollis*			279457	Extant
Cricetidae	Sigmodontinae	*Akodon mollis*			513601	Extant
Cricetidae	Sigmodontinae	*Akodon montensis*			121383	Extant
Cricetidae	Sigmodontinae	*Akodon montensis*			121386	Extant
Cricetidae	Sigmodontinae	*Akodon neocenus*			364531	Extant
Cricetidae	Sigmodontinae	*Akodon orophilus*			181336	Extant
Cricetidae	Sigmodontinae	*Akodon sanctipaulensis*			462077	Extant
Cricetidae	Sigmodontinae	*Akodon sanctipaulensis*			485113	Extant
Cricetidae	Sigmodontinae	*Akodon simulator*			259273	Extant
Cricetidae	Sigmodontinae	*Akodon spegazzinnii*			259279	Extant
Cricetidae	Sigmodontinae	*Akodon spegazzinnii*			259280	Extant
Cricetidae	Sigmodontinae	*Akodon subfuscus*			194762	Extant
Cricetidae	Sigmodontinae	*Akodon surdus*			194641	Extant
Cricetidae	Sigmodontinae	*Akodon surdus*			194642	Extant
Cricetidae	Sigmodontinae	*Akodon toba*			390148	Extant
Cricetidae	Sigmodontinae	*Akodon toba*			555664	Extant
Cricetidae	Sigmodontinae	*Akodon torques*			194610	Extant
Cricetidae	Sigmodontinae	*Akodon varius*			290908	Extant

Collection number and references of the extant and extinct rodent used in this work. *indicates the specimens for which we take the photograph in the Museum collections (Musée National d'Histoire Naturelle, Paris; American Museum of Natural History, New York; National Museum of Natural History, Washington DC).

Family	Subfamily	*Species*	References	Fossil site	Specimen ID	Extant/ Extinct
Cricetidae	Sigmodontinae	*Andalgalomys pearsoni*			262346	Extant
Cricetidae	Sigmodontinae	*Andinomys edax*			249021	Extant
Cricetidae	Sigmodontinae	*Andinomys edax*			541804	Extant
Cricetidae	Sigmodontinae	*Auliscomys boliviensis*			262772	Extant
Cricetidae	Sigmodontinae	*Auliscomys pictus*			16502	Extant
Cricetidae	Sigmodontinae	*Auliscomys pictus*			0	Extant
Cricetidae	Sigmodontinae	*Blarinomys breviceps*			304577	Extant
Cricetidae	Sigmodontinae	*Calomys callosus*			364803	Extant
Cricetidae	Sigmodontinae	*Calomys callosus*			364804	Extant
Cricetidae	Sigmodontinae	*Calomys callosus*			41756	Extant
Cricetidae	Sigmodontinae	*Calomys hummelineki*			460439	Extant
Cricetidae	Sigmodontinae	*Chelemys macronyx*			84230	Extant
Cricetidae	Sigmodontinae	*Chelemys macronyx*			84260	Extant
Cricetidae	Sigmodontinae	*Chelemys macronyx*			0	Extant
Cricetidae	Sigmodontinae	*Chilomys instans*			442342	Extant
Cricetidae	Sigmodontinae	*Chilomys instans*			579558	Extant
Cricetidae	Sigmodontinae	*Chilomys instans*			62922	Extant
Cricetidae	Sigmodontinae	*Chinchillula sahamae*			391816	Extant
Cricetidae	Sigmodontinae	*Delomys dorsalis*			484219	Extant
Cricetidae	Sigmodontinae	*Eligmodontia morgani*			21703	Extant
Cricetidae	Sigmodontinae	*Eligmodontia morgani*			236311	Extant
Cricetidae	Sigmodontinae	*Eligmodontia morgani*			236312	Extant

Collection number and references of the extant and extinct rodent used in this work. *indicates the specimens for which we take the photograph in the Museum collections (Musée National d'Histoire Naturelle, Paris; American Museum of Natural History, New York; National Museum of Natural History, Washington DC).

**Family**	**Subfamily**	***Species***	**References**	**Fossil site**	**Specimen ID**	**Extant/ Extinct**
Cricetidae	Sigmodontinae	*Eligmodontia typus*			262813	Extant
Cricetidae	Sigmodontinae	*Eligmodontia typus*			541732	Extant
Cricetidae	Sigmodontinae	*Euneomys mordax*			399400	Extant
Cricetidae	Sigmodontinae	*Euneomys mordax*			399401	Extant
Cricetidae	Sigmodontinae	*Geoxus valdivianus*			4	Extant
Cricetidae	Sigmodontinae	*Graomys domorum*			290923	Extant
Cricetidae	Sigmodontinae	*Graomys domorum*			38706	Extant
Cricetidae	Sigmodontinae	*Graomys domorum*			276847	Extant
Cricetidae	Sigmodontinae	*Graomys griseoflavus*			41844	Extant
Cricetidae	Sigmodontinae	*Handleyomys fuscatus*			32237	Extant
Cricetidae	Sigmodontinae	*Handleyomys fuscatus*			507267	Extant
Cricetidae	Sigmodontinae	*Holochilus brasiliensis*			41594	Extant
Cricetidae	Sigmodontinae	*Holochilus sciureus*			319980	Extant
Cricetidae	Sigmodontinae	*Ichthyomys hydrobates*			46729	Extant
Cricetidae	Sigmodontinae	*Ichthyomys hydrobates*			123323	Extant
Cricetidae	Sigmodontinae	*Ichthyomys hydrobates*			513625	Extant
Cricetidae	Sigmodontinae	*Ichthyomys stolzmanni*			10109	Extant
Cricetidae	Sigmodontinae	*Irenomys tarsalis*			391815	Extant
Cricetidae	Sigmodontinae	*Kunsia tomentosus*			584515	Extant
Cricetidae	Sigmodontinae	*Lenoxus apicalis*			16066	Extant
Cricetidae	Sigmodontinae	*Lenoxus apicalis*			72611	Extant
Cricetidae	Sigmodontinae	*Loxodontomys micropus*			84297	Extant

Collection number and references of the extant and extinct rodent used in this work. *indicates the specimens for which we take the photograph in the Museum collections (Musée National d'Histoire Naturelle, Paris; American Museum of Natural History, New York; National Museum of Natural History, Washington DC).

**Family**	**Subfamily**	***Species***	**References**	**Fossil site**	**Specimen ID**	**Extant/ Extinct**
Cricetidae	Sigmodontinae	*Lundomys molitor*			206388	Extant
Cricetidae	Sigmodontinae	*Megalomys desmarestii*			979385	Extant
Cricetidae	Sigmodontinae	*Melanomys caliginosus*			29533	Extant
Cricetidae	Sigmodontinae	*Melanomys caliginosus*			337765	Extant
Cricetidae	Sigmodontinae	*Melanomys caliginosus*			337583	Extant
Cricetidae	Sigmodontinae	*Microryzomys altissimus*			259587	Extant
Cricetidae	Sigmodontinae	*Microryzomys altissimus*			61922	Extant
Cricetidae	Sigmodontinae	*Microryzomys minutus*			303848	Extant
Cricetidae	Sigmodontinae	*Microryzomys minutus*			303849	Extant
Cricetidae	Sigmodontinae	*Microryzomys minutus*			67550	Extant
Cricetidae	Sigmodontinae	*Neacomys spinosus*			67525	Extant
Cricetidae	Sigmodontinae	*Neacomys tenuipes*			499542	Extant
Cricetidae	Sigmodontinae	*Neacomys tenuipes*			499541	Extant
Cricetidae	Sigmodontinae	*Necromys lasiurus*			290910	Extant
Cricetidae	Sigmodontinae	*Nectomys apicalis*			544406	Extant
Cricetidae	Sigmodontinae	*Nectomys apicalis*			544409	Extant
Cricetidae	Sigmodontinae	*Neotomys ebriosus*			541802	Extant
Cricetidae	Sigmodontinae	*Nesoryzomys darwini*			99937	Extant
Cricetidae	Sigmodontinae	*Nesoryzomys indefessus*			115831	Extant
Cricetidae	Sigmodontinae	*Neusticomys venezuelae*			406123	Extant
Cricetidae	Sigmodontinae	*Notiomys edwarsii*			1868269	Extant
Cricetidae	Sigmodontinae	*Oecomys auyantepui*			394247	Extant

Collection number and references of the extant and extinct rodent used in this work. *indicates the specimens for which we take the photograph in the Museum collections (Musée National d'Histoire Naturelle, Paris; American Museum of Natural History, New York; National Museum of Natural History, Washington DC).

**Family**	**Subfamily**	***Species***	**References**	**Fossil site**	**Specimen ID**	**Extant/ Extinct**
Cricetidae	Sigmodontinae	*Oecomys auyantepui*			394248	Extant
Cricetidae	Sigmodontinae	*Oecomys flavicans*			23625	Extant
Cricetidae	Sigmodontinae	*Oecomys superans*			68193	Extant
Cricetidae	Sigmodontinae	*Oligoryzomys andinus*			230984	Extant
Cricetidae	Sigmodontinae	*Oligoryzomys arenalis*			73204	Extant
Cricetidae	Sigmodontinae	*Oligoryzomys eliurus*			141454	Extant
Cricetidae	Sigmodontinae	*Oligoryzomys eliurus*			141448	Extant
Cricetidae	Sigmodontinae	*Oligoryzomys vegetus*			138078	Extant
Cricetidae	Sigmodontinae	*Oryzomys albigularis*			31430	Extant
Cricetidae	Sigmodontinae	*Oryzomys albigularis*			96163	Extant
Cricetidae	Sigmodontinae	*Oryzomys alfaroi*			32867	Extant
Cricetidae	Sigmodontinae	*Oryzomys alfaroi*			338254	Extant
Cricetidae	Sigmodontinae	*Oryzomys alfaroi*			338259	Extant
Cricetidae	Sigmodontinae	*Oryzomys alfaroi*			71596	Extant
Cricetidae	Sigmodontinae	*Oryzomys angouya*			248411	Extant
Cricetidae	Sigmodontinae	*Oryzomys angouya*			460270	Extant
Cricetidae	Sigmodontinae	*Oryzomys balneator*			513570	Extant
Cricetidae	Sigmodontinae	*Oryzomys chapmani*			71416	Extant
Cricetidae	Sigmodontinae	*Oryzomys couesi*			145021	Extant
Cricetidae	Sigmodontinae	*Oryzomys couesi*			82235	Extant
Cricetidae	Sigmodontinae	*Oryzomys couesi*			82237	Extant
Cricetidae	Sigmodontinae	*Oryzomys couesi*			82238	Extant

Collection number and references of the extant and extinct rodent used in this work. *indicates the specimens for which we take the photograph in the Museum collections (Musée National d'Histoire Naturelle, Paris; American Museum of Natural History, New York; National Museum of Natural History, Washington DC).

Family	Subfamily	*Species*	References	Fossil site	Specimen ID	Extant/ Extinct
Cricetidae	Sigmodontinae	*Oryzomys couesi*			82239	Extant
Cricetidae	Sigmodontinae	*Oryzomys couesi*			82241	Extant
Cricetidae	Sigmodontinae	*Oryzomys galapagoensis*			392255	Extant
Cricetidae	Sigmodontinae	*Oryzomys keaysi*			172972	Extant
Cricetidae	Sigmodontinae	*Oryzomys keaysi*			172972	Extant
Cricetidae	Sigmodontinae	*Oryzomys keaysi*			91532	Extant
Cricetidae	Sigmodontinae	*Oryzomys maracajuensis*			364747	Extant
Cricetidae	Sigmodontinae	*Oryzomys melanotis*			45318	Extant
Cricetidae	Sigmodontinae	*Oryzomys melanotis*			45319	Extant
Cricetidae	Sigmodontinae	*Oryzomys melanotis*			45326	Extant
Cricetidae	Sigmodontinae	*Oryzomys nelsoni*			89202	Extant
Cricetidae	Sigmodontinae	*Oryzomys nelsoni*			89203	Extant
Cricetidae	Sigmodontinae	*Oryzomys nitidus*			260370	Extant
Cricetidae	Sigmodontinae	*Oryzomys palustris*			23499	Extant
Cricetidae	Sigmodontinae	*Oryzomys palustris*			23500	Extant
Cricetidae	Sigmodontinae	*Oryzomys palustris*			23501	Extant
Cricetidae	Sigmodontinae	*Oryzomys palustris*			23502	Extant
Cricetidae	Sigmodontinae	*Oryzomys palustris*			23503	Extant
Cricetidae	Sigmodontinae	*Oryzomys palustris*			97775	Extant
Cricetidae	Sigmodontinae	*Oryzomys perenensis*			545298	Extant
Cricetidae	Sigmodontinae	*Oryzomys rhabdops*			76812	Extant
Cricetidae	Sigmodontinae	*Oryzomys rostratus*			108135	Extant

Collection number and references of the extant and extinct rodent used in this work. *indicates the specimens for which we take the photograph in the Museum collections (Musée National d'Histoire Naturelle, Paris; American Museum of Natural History, New York; National Museum of Natural History, Washington DC).

Family	Subfamily	*Species*	References	Fossil site	Specimen ID	Extant/ Extinct
Cricetidae	Sigmodontinae	*Oryzomys rostratus*			108137	Extant
Cricetidae	Sigmodontinae	*Oryzomys russatus*			542922	Extant
Cricetidae	Sigmodontinae	*Oryzomys russatus*			542922	Extant
Cricetidae	Sigmodontinae	*Oryzomys saturatior*			265085	Extant
Cricetidae	Sigmodontinae	*Oryzomys saturatior*			76358	Extant
Cricetidae	Sigmodontinae	*Oryzomys sp.*			98257	Extant
Cricetidae	Sigmodontinae	*Oryzomys talamancae*			306954	Extant
Cricetidae	Sigmodontinae	*Oryzomys xanthaeolus*			73131	Extant
Cricetidae	Sigmodontinae	*Oxymycterus amazonicus*			546010	Extant
Cricetidae	Sigmodontinae	*Oxymycterus amazonicus*			546015	Extant
Cricetidae	Sigmodontinae	*Paralomys gerbillus*			121142	Extant
Cricetidae	Sigmodontinae	*Phyllotis darwini*			541757	Extant
Cricetidae	Sigmodontinae	*Phyllotis darwini*			541758	Extant
Cricetidae	Sigmodontinae	*Phyllotis darwini*			93288	Extant
Cricetidae	Sigmodontinae	*Phyllotis wolffsohni*			260937	Extant
Cricetidae	Sigmodontinae	*Phyllotis xanthopygus*			260761	Extant
Cricetidae	Sigmodontinae	*Podoxymys roraimae*			75584	Extant
Cricetidae	Sigmodontinae	*Pseudoryzomys simplex*			262048	Extant
Cricetidae	Sigmodontinae	*Pseudoryzomys simplex*			364749	Extant
Cricetidae	Sigmodontinae	*Pseudoryzomys simplex*			584585	Extant
Cricetidae	Sigmodontinae	*Punomys lemminus*			116195	Extant
Cricetidae	Sigmodontinae	*Reithrodon auritus*			84205	Extant

Collection number and references of the extant and extinct rodent used in this work. *indicates the specimens for which we take the photograph in the Museum collections (Musée National d'Histoire Naturelle, Paris; American Museum of Natural History, New York; National Museum of Natural History, Washington DC).

**Family**	**Subfamily**	***Species***	**References**	**Fossil site**	**Specimen ID**	**Extant/ Extinct**
Cricetidae	Sigmodontinae	*Reithrodon auritus*			84208	Extant
Cricetidae	Sigmodontinae	*Reithrodon auritus*			235974	Extant
Cricetidae	Sigmodontinae	*Rheomys mexicanus*			205313	Extant
Cricetidae	Sigmodontinae	*Rhipidomys couesi*			516939	Extant
Cricetidae	Sigmodontinae	*Rhipidomys couesi*			516940	Extant
Cricetidae	Sigmodontinae	*Rhipidomys couesi*			559405	Extant
Cricetidae	Sigmodontinae	*Rhipidomys couesi*			559408	Extant
Cricetidae	Sigmodontinae	*Rhipidomys leucodactylus*			213199	Extant
Cricetidae	Sigmodontinae	*Rhipidomys leucodactylus*			63858	Extant
Cricetidae	Sigmodontinae	*Scapteromys aquaticus*			8379	Extant
Cricetidae	Sigmodontinae	*Scapteromys tumidus*			235431	Extant
Cricetidae	Sigmodontinae	*Scolomys melanops*			513581	Extant
Cricetidae	Sigmodontinae	*Scolomys melanops*			513582	Extant
Cricetidae	Sigmodontinae	*Scolomys melanops*			67522	Extant
Cricetidae	Sigmodontinae	*Sigmodon alleni*			88234	Extant
Cricetidae	Sigmodontinae	*Sigmodon alleni*			88235	Extant
Cricetidae	Sigmodontinae	*Sigmodon alleni*			26299	Extant
Cricetidae	Sigmodontinae	*Sigmodon alstoni*			415025	Extant
Cricetidae	Sigmodontinae	*Sigmodon alstoni*			415036	Extant
Cricetidae	Sigmodontinae	*Sigmodon alstoni*			75395	Extant
Cricetidae	Sigmodontinae	*Sigmodon arizonae*			91400	Extant
Cricetidae	Sigmodontinae	*Sigmodon arizonae*			96270	Extant

Collection number and references of the extant and extinct rodent used in this work. *indicates the specimens for which we take the photograph in the Museum collections (Musée National d'Histoire Naturelle, Paris; American Museum of Natural History, New York; National Museum of Natural History, Washington DC).

Family	Subfamily	*Species*	References	Fossil site	Specimen ID	Extant/ Extinct
Cricetidae	Sigmodontinae	*Sigmodon arizonae*			137004	Extant
Cricetidae	Sigmodontinae	*Sigmodon fulviventer*			21714	Extant
Cricetidae	Sigmodontinae	*Sigmodon fulviventer*			21715	Extant
Cricetidae	Sigmodontinae	*Sigmodon hispidus*			166599	Extant
Cricetidae	Sigmodontinae	*Sigmodon hispidus*			297921	Extant
Cricetidae	Sigmodontinae	*Sigmodontomys alfari*			28547	Extant
Cricetidae	Sigmodontinae	*Sigmodontomys alfari*			279744	Extant
Cricetidae	Sigmodontinae	*Thomasomys aureus*			251967	Extant
Cricetidae	Sigmodontinae	*Thomasomys aureus*			251976	Extant
Cricetidae	Sigmodontinae	*Thomasomys baeops*			948387	Extant
Cricetidae	Sigmodontinae	*Thomasomys cinereus*			73127	Extant
Cricetidae	Sigmodontinae	*Wiedomys pyrrhorhinos*			555760	Extant
Cricetidae	Sigmodontinae	*Wiedomys pyrrhorhinos*			555761	Extant
Cricetidae	Sigmodontinae	*Wilfredomys oenax*			206018	Extant
Cricetidae	Sigmodontinae	*Zygodontomys brevicauda*			461124	Extant
Cricetidae	Sigmodontinae	*Zygodontomys brevicauda*			75346	Extant
Cricetidae	Sigmodontinae	*Zygodontomys brevicauda*			85566	Extant
Cricetidae	Tylomyinae	*Nyctomys sumichrasti*			129892	Extant
Cricetidae	Tylomyinae	*Otonyctomys hatti*			91189	Extant
Cricetidae	Tylomyinae	*Ototylomys phyllotis*			79304	Extant
Cricetidae	Tylomyinae	*Tylomys watsoni*			18775	Extant
Cricetidae	Tylomyinae	*Tylomys watsoni*			464884	Extant

Collection number and references of the extant and extinct rodent used in this work. *indicates the specimens for which we take the photograph in the Museum collections (Musée National d'Histoire Naturelle, Paris; American Museum of Natural History, New York; National Museum of Natural History, Washington DC).

**Family**	**Subfamily**	***Species***	**References**	**Fossil site**	**Specimen ID**	**Extant/ Extinct**
Cricetidae	Tylomyinae	*Tylomys watsoni*			464885	Extant
Cricetidae		*Apocricetus aff. plinii*	Freudenthal et al., 1998		RGM 413 877	Fossil
Cricetidae		*Apocricetus aff. plinii*	Freudenthal et al., 1998		RGM 413 880	Fossil
Cricetidae		*Apocricetus alberti*	de Bruijn et al., 1975	*Crevillente 6*	CR6 162	Fossil
Cricetidae		*Apocricetus alberti*	Adrover et al., 1993	La Gloria 5	LG5 16	Fossil
Cricetidae		*Apocricetus barrierei*	Martín-Suárez, 1988	Botardo C	Bo-C 17	Fossil
Cricetidae		*Apocricetus barrierei*	Chaline, 1984	Chabrier	CHA 65356	Fossil
Cricetidae		*Apocricetus barrierei*	Ruíz Bustos et al., 1984		G 32	Fossil
Cricetidae		*Apocricetus barrierei*	Freudenthal et al., 1998	Purcal 4	PUR-4 14	Fossil
Cricetidae		*Apocricetus barrierei*	García-Alix, 2006; García-Alix et a., 2008b	Purcal 4	PUR-4 4	Fossil
Cricetidae		*Apocricetus plinii*	Freudenthal et al., 1991	Crevillente 15	RGM 402 160	Fossil
Cricetidae		*Apocricetus plinii*	Freudenthal et al., 1991	Crevillente 15	RGM 402 171	Fossil
Cricetidae		*Blancomys sanzi*	Adrover et al., 1993	La Gloria 5	LG5 33	Fossil
Cricetidae		*Blancomys sanzi*	Adrover et al., 1993	La Gloria 5	LG5 34	Fossil

Collection number and references of the extant and extinct rodent used in this work. *indicates the specimens for which we take the photograph in the Museum collections (Musée National d'Histoire Naturelle, Paris; American Museum of Natural History, New York; National Museum of Natural History, Washington DC).

**Family**	**Subfamily**	***Species***	**References**	**Fossil site**	**Specimen ID**	**Extant/ Extinct**
Cricetidae		*Blancomys sanzi*	García-Alix, 2006; García-Alix et a., 2008b	Purcal-4	PUR-4 781	Fossil
Cricetidae		*Cricetodon albanensis*	Mein & Freudenthal, 1971	La Grive-Saint Alban (fente M)	FSL 65485	Fossil
Cricetidae		*Cricetodon cf. jotae*	Sesé, 2004		BA-27	Fossil
Cricetidae		*Cricetodon jotae*	Antunes & Mein, 1977		Fig. 45	Fossil
Cricetidae		*Cricetulodon bugesiensis*	Freudenthal et al., 1998		FSL 65903	Fossil
Cricetidae		*Cricetulodon bugesiensis*	Freudenthal et al., 1998		FSL 65904	Fossil
Cricetidae		*Cricetulodon bugesiensis*	Freudenthal et al., 1998		FSL 65925	Fossil
Cricetidae		*Cricetulodon meini*	Agustí, 1986		CA-1	Fossil
Cricetidae		*Cricetulodon meini*	Agustí, 1986		CA-2	Fossil
Cricetidae		*Cricetulodon meini*	Agustí, 1986		CA-20	Fossil
Cricetidae		*Cricetulodon sabadellensis*	Agustí 1981	Can Llobateres		Fossil
Cricetidae		*Cricetulodon sabadellensis*	Agustí 1981	Can Llobateres		Fossil
Cricetidae		*Democricetodon brevis*	Casanovas-Vilar, 2007; Casanovas-Vilar et al., 2010		IPS 23096	Fossil

Collection number and references of the extant and extinct rodent used in this work. *indicates the specimens for which we take the photograph in the Museum collections (Musée National d'Histoire Naturelle, Paris; American Museum of Natural History, New York; National Museum of Natural History, Washington DC).

**Family**	**Subfamily**	***Species***	**References**	**Fossil site**	**Specimen ID**	**Extant/ Extinct**
Cricetidae		*Democricetodon brevis*	Peláez-Campomanes & Daams, 2000	Palasar	PSL151	Fossil
Cricetidae		*Democricetodon brevis*	Peláez-Campomanes & Daams, 2000	Palasar	PSL152	Fossil
Cricetidae		*Democricetodon brevis*	Peláez-Campomanes & Daams, 2000	Palasar	PSL155	Fossil
Cricetidae		*Democricetodon cf. brevis*	Aguilar et al., 1979	Castell de Barbera	CBBp4fig10	Fossil
Cricetidae		*Democricetodon cf. brevis*	Aguilar et al., 1979	Castell de Barbera	CBBp4fig11	Fossil
Cricetidae		*Democricetodon cf. brevis*	Aguilar et al., 1979	Castell de Barbera	CBBp4fig12	Fossil
Cricetidae		*Democricetodon cf. brevis*	Aguilar et al., 1979	Castell de Barbera	CBBp4fig14	Fossil
Cricetidae		*Democricetodon cf. crusafonti*	Freudenthal & Daams, 1988		RGM 301 517	Fossil
Cricetidae		*Democricetodon cf. crusafonti*	Freudenthal & Daams, 1988		RGM 301 519	Fossil
Cricetidae		*Democricetodon cf. gracilis*	Aguilar et al., 2010	Blanquatère 3	BLQ3 66	Fossil
Cricetidae		*Democricetodon crusafonti*	Freudenthal & Daams, 1988		RGM 268 408	Fossil
Cricetidae		*Democricetodon crusafonti*	Lacomba, 1988		RGM 336 906	Fossil
Cricetidae		*Democricetodon larteti*	Casanovas-Vilar, 2007; Casanovas-Vilar et al., 2010		IPS 23260	Fossil

Collection number and references of the extant and extinct rodent used in this work. *indicates the specimens for which we take the photograph in the Museum collections (Musée National d'Histoire Naturelle, Paris; American Museum of Natural History, New York; National Museum of Natural History, Washington DC).

**Family**	**Subfamily**	***Species***	**References**	**Fossil site**	**Specimen ID**	**Extant/ Extinct**
Cricetidae		*Democricetodon larteti*	Casanovas-Vilar, 2007; Casanovas-Vilar et al., 2010		IPS 23308	Fossil
Cricetidae		*Democricetodon larteti*	Freudenthal & Daams, 1988		RGM 252 506	Fossil
Cricetidae		*Democricetodon larteti*	Freudenthal & Daams, 1988		RGM 252 519	Fossil
Cricetidae		*Democricetodon larteti*	Freudenthal & Daams, 1988		RGM 268 116	Fossil
Cricetidae		*Democricetodon larteti*	Freudenthal & Daams, 1988		RGM 268 121	Fossil
Cricetidae		*Democricetodon larteti*	Freudenthal & Daams, 1988		RGM 268 122	Fossil
Cricetidae		*Democricetodon larteti*	Freudenthal & Daams, 1988		RGM 268 138	Fossil
Cricetidae		*Democricetodon sulcatus*	Lacomba, 1988		RGM 337 081	Fossil
Cricetidae		*Eumyarion cf medius*	Prieto & Rumel, 2009	Petersbech 68	NMA 2007-149/2017	Fossil
Cricetidae		*Eumyarion leemani*	Aguilar et al., 1979	Castell de Barbera		Fossil
Cricetidae		*Eumyarion leemani*	Aguilar et al., 1979	Castell de Barbera		Fossil
Cricetidae		*Eumyarion leemani*	Aguilar et al., 1979	Castell de Barbera		Fossil

Collection number and references of the extant and extinct rodent used in this work. *indicates the specimens for which we take the photograph in the Museum collections (Musée National d'Histoire Naturelle, Paris; American Museum of Natural History, New York; National Museum of Natural History, Washington DC).

Family	Subfamily	*Species*	References	Fossil site	Specimen ID	Extant/ Extinct
Cricetidae		*Eumyarion leemani*	Casanovas-Vilar, 2007; Casanovas-Vilar et al., 2010		IPS 23124	Fossil
Cricetidae		*Eumyarion medius*	Kälin, 1999	Sansan		Fossil
Cricetidae		*Hispanomys adroveri*	Agustí, 1986	Collado Villalta		Fossil
Cricetidae		*Hispanomys adroveri*	Agustí, 1986		FCA-152	Fossil
Cricetidae		*Hispanomys adroveri*	Freudenthal et al., 1991	Crevillente 15	RGM 402 245	Fossil
Cricetidae		*Hispanomys adroveri*	Freudenthal et al., 1991	Crevillente 15	RGM 402 247	Fossil
Cricetidae		*Hispanomys aguirrei*	Sesé, 1980	Escobosa de Calatañazor	ES-221	Fossil
Cricetidae		*Hispanomys aguirrei*	Sesé, 1980	Escobosa de Calatañazor	ES-715	Fossil
Cricetidae		*Hispanomys cf. aguirrei*	Casanovas-Vilar, 2007; Casanovas-Vilar et al., 2010		IPS 23285	Fossil
Cricetidae		*Hispanomys cf. aragoniensis*	Antunes et al., 1983			Fossil
Cricetidae		*Hispanomys cf. aragoniensis*	Antunes et al., 1983			Fossil
Cricetidae		*Hispanomys cf. thaleri*	Aguilar et al., 1979	Castell de Barbera		Fossil
Cricetidae		*Hispanomys daamsi*	Agustí et al., 2005	Can Missert		Fossil
Cricetidae		*Hispanomys dispectus*	Agustí, 1980	Castell de Barbera	CBB	Fossil

Collection number and references of the extant and extinct rodent used in this work. *indicates the specimens for which we take the photograph in the Museum collections (Musée National d'Histoire Naturelle, Paris; American Museum of Natural History, New York; National Museum of Natural History, Washington DC).

**Family**	**Subfamily**	***Species***	**References**	**Fossil site**	**Specimen ID**	**Extant/ Extinct**
Cricetidae		*Hispanomys freudenthali*	Adrover, 1986	Los Aguanaces	LA 542	Fossil
Cricetidae		*Hispanomys freudenthali*	van der Weerd, 1976	Masada del Valle 2	MDV2 1241	Fossil
Cricetidae		*Hispanomys freudenthali*	Alcalá et al., 1990	Puente Minero	PM-697	Fossil
Cricetidae		*Hispanomys freudenthali*	Alcalá et al., 1990	Puente Minero	PM-701	Fossil
Cricetidae		*Hispanomys mediterraneus*	Farjanel & Mein, 1984	Ambérieu		Fossil
Cricetidae		*Hispanomys moralesi*	López-Antoñanzas et al., 2010	Batallones 10	BAT10'07-F4-9	Fossil
Cricetidae		*Hispanomys moralesi*	López-Antoñanzas et al., 2010	Batallones 10	BAT10'07-G3-23a	Fossil
Cricetidae		*Hispanomys moralesi*	López-Antoñanzas et al., 2010	Batallones 10	BAT10'07-G3-23b	Fossil
Cricetidae		*Hispanomys moralesi*	López-Antoñanzas et al., 2010	Batallones 1	BAT1M'02-D7-2	Fossil
Cricetidae		*Hispanomys moralesi*	López-Antoñanzas et al., 2010	Batallones 1	BAT1M'02-E5-1	Fossil
Cricetidae		*Hispanomys moralesi*	López-Antoñanzas et al., 2010	Batallones 1	BAT1M'03-D3-4	Fossil
Cricetidae		*Hispanomys nombrevillae*	Lacomba, 1988		RGM 336 697	Fossil
Cricetidae		*Hispanomys peralensis*	van der Weerd, 1976	Peralejos C	PERC 351	Fossil
Cricetidae		*Hispanomys peralensis*	Freudenthal et al., 1991	Crevillente 2	*RGM 402 292*	Fossil

Collection number and references of the extant and extinct rodent used in this work. *indicates the specimens for which we take the photograph in the Museum collections (Musée National d'Histoire Naturelle, Paris; American Museum of Natural History, New York; National Museum of Natural History, Washington DC).

**Family**	**Subfamily**	***Species***	**References**	**Fossil site**	**Specimen ID**	**Extant/ Extinct**
Cricetidae		*Hispanomys peralensis*	Freudenthal et al., 1991	Crevillente 2	RGM 404 864	Fossil
Cricetidae		*Hispanomys thaleri*	Agusti & Gibert 1982	Can Perellada		Fossil
Cricetidae		*Hispanomys thaleri*	Agustí 1981	Can Perellada		Fossil
Cricetidae		*Megacricetodon aff. fournasi*	Aguilar et al., 2010b		PUI 13	Fossil
Cricetidae		*Megacricetodon aff. fournasi*	Aguilar et al., 2010b		PUI 15	Fossil
Cricetidae		*Megacricetodon cf. debruijni*	Aguilar et al., 1979	Castell de Barbera		Fossil
Cricetidae		*Megacricetodon cf. ibericus*	Agustí et al., 2005	Can Missert		Fossil
Cricetidae		*Megacricetodon cf. ibericus*	Agustí et al., 2005	Can Missert		Fossil
Cricetidae		*Megacricetodon crusafonti*	Antunes & Mein, 1977			Fossil
Cricetidae		*Megacricetodon debruijni*	Álvarez-Sierra & García Moreno, 1986	Ampudia 9	AMP9-RH1	Fossil
Cricetidae		*Megacricetodon debruijni*	Álvarez-Sierra & García Moreno, 1986	Ampudia 9	AMP9-RH15	Fossil
Cricetidae		*Megacricetodon debruijni*	Álvarez-Sierra & García Moreno, 1986	Ampudia 9	AMP9-RH2	Fossil
Cricetidae		*Megacricetodon fournasi*	Aguilar, 1995	Lo Fournas 2	FOU2 186	Fossil
Cricetidae		*Megacricetodon fournasi*	Aguilar, 1995	Lo Fournas 2	FOU2 187	Fossil
Cricetidae		*Megacricetodon gr. minor-debruijni*	Sesé & López Martínez, 1981			Fossil

Collection number and references of the extant and extinct rodent used in this work. *indicates the specimens for which we take the photograph in the Museum collections (Musée National d'Histoire Naturelle, Paris; American Museum of Natural History, New York; National Museum of Natural History, Washington DC).

Family	Subfamily	*Species*	References	Fossil site	Specimen ID	Extant/ Extinct
Cricetidae		*Megacricetodon ibericus*		Escobosa de Calatañazor	ES-611	Fossil
Cricetidae		*Megacricetodon ibericus*	Antunes et al., 1983			Fossil
Cricetidae		*Megacricetodon ibericus*	Agustí 1981	Hostalets de Pierola superior		Fossil
Cricetidae		*Megacricetodon ibericus*	Lacomba, 1988		RGM 336 979	Fossil
Cricetidae		*Megacricetodon ibericus*	Lacomba, 1988		RGM 337 015	Fossil
Cricetidae		*Megacricetodon lemartinelli*	Aguilar, 1995	Lo Fournas 10	FOU10 101	Fossil
Cricetidae		*Megacricetodon lemartinelli*	Aguilar, 1995	Lo Fournas 10	FOU10 107	Fossil
Cricetidae		*Megacricetodon lemartinelli*	Aguilar, 1995	Lo Fournas 10	FOU10 110	Fossil
Cricetidae		*Megacricetodon lopezae*	Álvarez-Sierra & García Moreno, 1986	Simancas 2	SIM2-RH3	Fossil
Cricetidae		*Megacricetodon lopezae*	Álvarez-Sierra & García Moreno, 1986	Simancas 2	SIM2-RH4	Fossil
Cricetidae		*Megacricetodon lopezae*	Álvarez-Sierra & García Moreno, 1986	Simancas 2	SIM2-RH5	Fossil
Cricetidae		*Megacricetodon minor*	Wessels & Reumer, 2009	Sandelzhausen	9220	Fossil
Cricetidae		*Megacricetodon minor*	Wessels & Reumer, 2009	Sandelzhausen	9221	Fossil
Cricetidae		*Megacricetodon minor*	Casanovas-Vilar, 2007		IPS 23283	Fossil

Collection number and references of the extant and extinct rodent used in this work. *indicates the specimens for which we take the photograph in the Museum collections (Musée National d'Histoire Naturelle, Paris; American Museum of Natural History, New York; National Museum of Natural History, Washington DC).

**Family**	**Subfamily**	***Species***	**References**	**Fossil site**	**Specimen ID**	**Extant/ Extinct**
Cricetidae		*Megacricetodon minor*	García Moreno, 1986	Valladolid 1	VA1-RH2	Fossil
Cricetidae		*Megacricetodon minor-debruijni*	Álvarez-Sierra, 1983	Torremormojón 3	TM3-2	Fossil
Cricetidae		*Megacricetodon minor-debruijni*	Álvarez-Sierra, 1983	Torremormojón 4	TM4-34	Fossil
Cricetidae		*Megacricetodon roussillonensis*	Aguilar, 1995	Lo Fournas 3	FOU3 139	Fossil
Cricetidae		*Megacricetodon roussillonensis*	Aguilar, 1995	Lo Fournas 3	FOU3 140	Fossil
Cricetidae		*Microtocicetus molassicus*	Hír & Kókay, 2010		FT 3/10	Fossil
Cricetidae		*Myocricetodon cf. M. parvus*	Wessels, 2009	H-GSP 82.24	H-GSP 82.24 - Fig 2.2-5	Fossil
Cricetidae		*Myocricetodon jaegeri*	Minwer-Barakat, 2005; Minwer-Barakat et al., 2009b	Negratín-1	NGR-1 88	Fossil
Cricetidae		*Myocricetodon jaegeri*	Minwer-Barakat, 2005; Minwer-Barakat et al., 2009b	Negratín-1	NGR-1 91	Fossil
Cricetidae		*Neocricetodon ambarrensis*	Freudenthal et al., 1998		FSL 65913	Fossil
Cricetidae		*Neocricetodon ambarrensis*	Freudenthal et al., 1998		FSL 65914	Fossil
Cricetidae		*Neocricetodon ambarrensis*	Freudenthal et al., 1998		FSL 65921	Fossil
Cricetidae		*Neocricetodon fahlbuschi*	Bachmayer & Wilson 1980			Fossil

Collection number and references of the extant and extinct rodent used in this work. *indicates the specimens for which we take the photograph in the Museum collections (Musée National d'Histoire Naturelle, Paris; American Museum of Natural History, New York; National Museum of Natural History, Washington DC).

**Family**	**Subfamily**	***Species***	**References**	**Fossil site**	**Specimen ID**	**Extant/ Extinct**
Cricetidae		*Neocricetodon lucentensis*	Freudenthal et al., 1991		RGM 404 675	Fossil
Cricetidae		*Neocricetodon lucentensis*	Freudenthal et al., 1991		RGM 404 686	Fossil
Cricetidae		*Neocricetodon occidentalis*	Freudenthal et al., 1991	Crevillente 2	RGM 385 782	Fossil
Cricetidae		*Neocricetodon occidentalis*	Freudenthal et al., 1991	Crevillente 2	RGM 385 787	Fossil
Cricetidae		*Neocricetodon seseae*	Freudenthal et al., 1998	Creu Conill 22	CR22 10	Fossil
Cricetidae		*Neocricetodon seseae*	Aguilar et al., 1995		CTN67	Fossil
Cricetidae		*Neocricetodon seseae*	Aguilar et al., 1995		CTN78	Fossil
Cricetidae		*Neocricetodon seseae*	Freudenthal et al., 1998		RGM 402 073	Fossil
Cricetidae		*Neocricetodon skofleki*	Freudenthal et al., 1998		FSL 65934	Fossil
Cricetidae		*Neocricetodon skofleki*	Freudenthal et al., 1998		FSL 65938	Fossil
Cricetidae		*Rotundomys bressanus*	Agustí & Gibert, 1982	Torrent de Febulines		Fossil
Cricetidae		*Rotundomys cf. montisrotundi*	Agustí 1981	Can Llobateres		Fossil
Cricetidae		*Rotundomys cf. mundi*	Agustí 1981	Can Perellada		Fossil

Collection number and references of the extant and extinct rodent used in this work. *indicates the specimens for which we take the photograph in the Museum collections (Musée National d'Histoire Naturelle, Paris; American Museum of Natural History, New York; National Museum of Natural History, Washington DC).

**Family**	**Subfamily**	***Species***	**References**	**Fossil site**	**Specimen ID**	**Extant/ Extinct**
Cricetidae		*Rotundomys montisrotundi*	Kälin, 1999	Montredon	Montredon	Fossil
Cricetidae		*Ruscinomys aff. schaubi*	García-Alix, 2006; García-Alix et a., 2008b	Dehesa 16	DHS-16 197	Fossil
Cricetidae		*Ruscinomys bravoi*	Adrover & Mein, 1996	Aljezar B	AB 3006	Fossil
Cricetidae		*Ruscinomys lasaliei*	Ruíz Bustos et al., 1984		G 19	Fossil
Cricetidae		*Ruscinomys lasaliei*	Adrover et al., 1993	La Gloria 4	LG4 8	Fossil
Cricetidae		*Ruscinomys lasaliei*	García-Alix, 2006; García-Alix et a., 2008b	Purcal 13	PUR-13 190	Fossil
Cricetidae		*Ruscinomys lasaliei*	García-Alix, 2006; García-Alix et a., 2008b	Purcal 4	PUR-4 832	Fossil
Cricetidae		*Ruscinomys schaubi*	Adrover, 1986	Aljezar B	AB 1423	Fossil
Cricetidae		*Ruscinomys schaubi*	Adrover, 1986	Aljezar B	AB 1464	Fossil
Cricetidae		*Ruscinomys schaubi*	Adrover, 1986	Aljezar B	AB 1485	Fossil
Cricetidae		*Ruscinomys schaubi*	van der Weerd, 1976	Los Mansuetos	LM 754	Fossil
Cricetidae		*Trilophomys castroi*	Adrover, 1986	ARR	ARR 285	Fossil
Cricetidae		*Trilophomys castroi*	Adrover, 1986	VAL	VAL 229	Fossil
Cricetidae		*Trilophomys cf. castroi*	Adrover, 1986	ORO	ORO 12	Fossil

Collection number and references of the extant and extinct rodent used in this work. *indicates the specimens for which we take the photograph in the Museum collections (Musée National d'Histoire Naturelle, Paris; American Museum of Natural History, New York; National Museum of Natural History, Washington DC).

**Family**	**Subfamily**	***Species***	**References**	**Fossil site**	**Specimen ID**	**Extant/ Extinct**
Cricetidae		*Trilophomys cf. castroi*	Adrover et al 1985	VAR	VAR 15a	Fossil
Cricetidae		*Trilophomys vanderweerdi*	Adrover, 1986	OR	OR 3 79	Fossil

References:

Adrover, R., Agusti, J., Moyá-Solá, S., Pons, J., 1985. Nueva localidad de micromamiferos insulares del mioceno medio en las proxi midades de San Lorenzo en la isla de Mallorca. PALEONTOLOGIA I EVOLUCIÓ 18, 121-129.

Adrover, R., 1986. Nuevas faunas de roedores en el Mio-Plioceno continental de la región de Teruel (España). Interés bioestratigráfico y paleoecológico. Instituto de Estudios Turolenses.

Adrover, R., Mein, P., Moissenet, E., 1993. Roedores de latransición Mio-Plioceno de la región de Teruel. Paleontologia i Evolució 26-27, 47-84.

Adrover, R., Mein, P., 1996. Nuevo Ruscinomys (Rodentia, Mammalia) en el Mioceno superior de la región de Teruel (España). Estudios Geológicos 52, 361-365.

Aguilar, J.-P., Agustí, J., Gibert, J., 1979. Rongeurs miocènes dans le Valles-Penedes. 2-Rongeurs de Castell de Barbera. Palaeovertebrata 9, 17–31.

Aguilar, J.-P., Michaux, J., Bachelet, B., Calvet, M., Faillat, J.-P., 1991. Les nouvelles faunes de rongeurs proches de la limite MioPliocène en Roussillon. Implications biostratigraphiques et biogéographiques. Palaeovertebrata 20, 147–174.

Aguilar, J.-P., 1995. Evolution de la lignee Megacricetodon collongensis-Megacricetodon rousillonensis (Cricetidae, Rodentia, Mammalia) au cours du Miocene inferieur et moyen dans le sud de la France. Palaeovertebrata 24, 1-23.

Aguilar, J.-P., Michaux, J., 1995. Chronologie mammalienne et grade-datations dans le Miocène inferieur et moyen français: une revue. Géologie de la France 1, 69-76.

Aguilar, J.-P., Michaux, J., 2010. Nouvelles Faunes de Rongeurs (Mammalia, Rodentia) d'âge Miocène Moyen en Languedoc-Roussillon (Sud de la France); Biostratigraphie et Corrélations. Geodiversitas.

Agusti, J., 1981. Roedores miomorfos del Neogeno de Cataluña. Universidad de Barcelona.

Agustí, J., 1980. La asociación de Hispanomys y Cricetodon (Rodentia, Mammalia) en el Mioceno Superior del Vallès-Penedès (Cataluña, España). Acta geológica hispánica 15, 51-60.

Agustí, J., Gibert, J., 1982. Roedores e Insectivoros del Mioceno superior dels hostalets de Pierola (Vallés-Penedes, Cataluña). Bull. Inf. Inst. Paleont. Sabadell 14, 18-37.

Agustí, J., 1986. Nouvelles espèces de Cricetidés vicariantesdans le Turolien moyen de Fortuna (Prov. Murcia, Espagne). Geobios 19, 5-16.

Agustí, J., Casanovas-Vilar, I., Furió, M., 2005. Rodents, insectivores and chiropterans (Mammalia) from the late Aragonian of Can Missert (Middle Miocene, Vallès-Penedès Basin, Spain). Geobios 38, 575-583.

Alcalá, L., Cerdeño, E., Montoya, P., Morales, J., Pérez, B., Soria, D., 1990. Composición taxonómica y anatómica de los restos de macrovertebrados del Mioceno inferior continental de Loranca del Campo (Cuenca). Comunicaciones de la Reunión de Tafonomía y Fosilización, 7-12.

Álvarez-Sierra, M.A., 1983. Paleontología y Bioestratigrafía del Mioceno superior del sector central de la cuenca del Duero. Estudio de los micromamíferos de la serie de Torremormojón (Palencia). Universidad Complutense de Madrid, Spain, p. 155.

Álvarez-Sierra, M.A., García Moreno, E., 1986. New Gliridae and Cricetidae (Mamm. Rod.) from the Middle and Upper Miocene of the Duero basin, Spain. Studia geologica salmanticensia, 145-189.

Antunes, M.T., Mein, P., 1977. Contributions à la paléontologie du Miocène Moyen Continental du Bassin du Tage III Mammifères - Povoa De Santarém, Pero Filhü Et Choes (Secorio) Conclusions Générales. Ciências da Terra 3, 143-165.

Antunes, M.T., Ginsburg, L., Mein, P., 1983. Mammifères miocènes de Azambujeira, niveau inférieur (Santarém, Portugal). Ciências da Terra 7, 161-186.

Antunes, M.T., Pais, J., 1992. The Neogene of Portugal.

Bachelet, B., 1990. Muridae et Arvicolidae (Rodentia, Mammalia) du Pliocene du Sud de la France: systematique, evolution, biochronologie. Univ. Montpellier, France, p. 180.

Bachmayer, F., Wilson, R.W., 1980. A third contribution to the fossil small mammal fauna of Kohfidisch (Burgenland), Austria. Annalen des Naturhistorischen Museums in Wien 83, 351-386.

Casanovas-Vilar, I., 2007. Universitat Autònoma de Barcelona, pp. 1-283.

Casanovas-Vilar, I., Van Dam, j.A., Moyà-Solà, S., Rook, L., 2011. Late Miocene insular mice from the Tusco-Sardinian palaeobioprovince provide new insights on the palaeoecology of the Oreopithecus faunas. Journal of Human Evolution 61, 42-49.

Casanovas-Vilar…, I., Angelone, C., Alba, D.M., Moyà-Solà, S., Köhler, M., Galindo, J., 2010. Rodents and lagomorphs from the Middle Miocene hominoid-bearing site of Barranc de Can Vila 1 (els Hostalets de Pierola, Catalonia, Spain). Neues Jahrbuch für Geologie und Paläontologie - Abhandlungen 257, 297-315.

Chaline, J., 1984. La sequence des rongeurs de Bresse, en tant que reference biostratigraphique et paleoclimatique. Geol. France 3, 251-268. .

Cooper, N.K., Adams, M., Anthony, C., 2003. Morphological and genetic variation in Leggadina (Thomas, 1910) with special reference to Western Australian

populations. RECORDS-WESTERN ….
de Bruijn, H., Mein, P., Montenat, C., van de Weerd, A., 1975. Correlations entre les gisements de rongeurs et les formations marines du Miocene terminal d'Espagne meridionale (prov. de Alicante et Murcia). Proc. Kon. Ned. Akad. Wetensch. B. 78, 282-313.
Durden, L.A., Musser, G.G., 1991. A new species of sucking louse (Insecta, Anoplura) from a montane forest rat in Central Sulawesi and a preliminary interpretation of the sucking louse fauna of Sulawesi. American Museum of Natural History.
Emmons, L.H., 1993. A new genus and species of rat from Borneo (Rodentia: Muridae). Proceedings of the Biological Society of Washington 106, 752-761.
Farjanel, G., Mein, P., 1984. Une association de mammifères et de pollens dans la formation continentale des "Marnes de Bresse" d'âge Miocène supérieur, à Ambérieu (Ain). Géologie de la France 1–2, 131–148.
Freudenthal, M., Daams, R., 1988. Cricetidae (Rodentia) from the type Aragonian; the genera Democricetodon, Fahlbuschia, Pseudofahlbuschia nov. gen., and Renzimys. Scripta Geologica. Special Issue.
Freudenthal, M., Lacomba, J.I., Martín-Suárez, E., 1991. The Cricetidae (Mammalia, Rodentia) from the Late Miocene of Crevillente (prov. Alicante, Spain). Scripta Geologica 96, 9-46.
Freudenthal, M., Mein, P., Martín-Suárez, E., 1998. Revision of Late Miocene and Pliocene Cricetinae (Rodentia, Mammalia) from Spain and France. Treballs del Museu de Geologia de Barcelona 7, 11-93.
Freudenthal, M., Martín-Suárez, E., 1999. Family Muridae, in: Rössner, G., Heissig, K. (Eds.), The Miocene land mammals of Europe. Verlang Dr. Friedrich Pfeil, München, pp. 401-409.
García Moreno, E., Álvarez-Sierra, M.A., 1986. New Gliridae and Cricetidae (Mamm. Rod.) from the Middle and Upper Miocene of the Duero basin, Spain. Studia geologica salmanticensia, 145-189.
García-Alix, A., 2006. Universidad de Granada, pp. 1-429.
García-Alix, A., Minwer-Barakat, R., Martín-Suárez, E., Freudenthal, M., 2008a. Cricetidae and Gliridae (Rodentia, Mammalia) from the Miocene and Pliocene and Southern Spain. Scripta Geologica 136, 1:37.
García-Alix, A., Minwer-Barakat, R., Suarez, E., Freudenthal, M., 2008b. Muridae (Rodentia, Mammalia) from the Mio-Pliocene boundary in the Granada Basin (southern Spain). Biostratigraphic and phylogenetic implications. Neues Jahrbuch f&# 252; r Geologie und Pal&# 228; ontologie-Abhandlungen 248, 183-215.
Guerra-Merchan, A., Ramallo, D., Ruiz Bustos, A., 2001. New data on the upper miocene micromammals of the Betic cordillera and their interest form

marine-continental correlations. Geobios 34, 85-90.
Helgen, K.M., 2005. The amphibious murines of New Guinea (Rodentia, Muridae): the generic status of Baiyankamys and description of a new species of Hydromys. Zootaxa 913, 1-20.
Helgen, K.M., Helgen, L.E., 2009. Chapter 8. Biodiversity and Biogeography of the Moss-mice of New Guinea: A Taxonomic Revision of Pseudohydromys (Muridae: Murinae). Bulletin of the American Museum of Natural History 331, 230-313.
Hír, J., Kókay, J., 2010. A systematic study of the middle-late miocene rodents and lagomorphs (Mammalia) of Felsőtárkány 3/8 and 3/10 (Northern Hungary) with stratigraphical relations. Geodiversitas 32, 307-329.
Kälin, D., 1999. Tribe Cricetini., in: Rössner, E., Heissig, K. (Eds.), The Miocene Land Mammals of Europe. Verlag Dr. Friedrich Pfeil, München, pp. 373-387.
Kaneko, Y., 2001. Morphological discrimination of the Ryukyu spiny rat (genus Tokudaia) between the islands of Okinawa and Amami Oshima, in the Ryukyu Islands, southern Japan. Mammal Study 26, 17-33.
Kitchener, D.J., Maryanto, I., 1995. A new species of Melomys (Rodentia, Muridae) from Yamdena Island, Tanimbar Group, Eastern Indonesia. Records of the Western Australian Museum 17, 43-50.
Lacomba, J.I., 1988. Rodents and lagomorphs from a lower Vallesian fissure filling near Molina de Aragón (prov. Guadalajara, Spain). Scripta Geologica Special Issue 1, 19-38.
Lazzari, V., Aguilar, J.-P., Michaux, J., 2010. Intraspecific variation and micro-macroevolution connection: illustration with the late Miocene genus Progonomys (Rodentia, Muridae). Paleobiology 36, 641-657.
López-Antoñanzas, R., Peláez-Campomanes, P., Álvarez-Sierra, M.A., García-Paredes, I., 2010. New species of Hispanomys (Rodentia, Cricetodontinae) from the Upper Miocene of Batallones (Madrid, Spain). Zoological Journal of the Linnean Society 160, 725-747.
Martin-Suárez, E., 1988. Une nouvelle espèce d'Apodemus (Rodentia, Mammalia) du Pliocène de la dépression de Guadix-Baza (Grenade, Espagne). Geobios 21, 523-529.
Martín-Suárez, E., Freudenthal, M., 1993. Muridae (Rodentia) from the Lower Turolian of Crevillente (Alicante, Spain). Scripta Geologica 103, 65-118.
Martín-Suárez, E., Freudenthal, M., 1994. Castromys, a new genus of Muridae (Rodentia) from the Late Miocene of Spain. Scripta Geologica 106, 11-34.
Mein, P., Freudenthal, M., 1971. Une nouvelle classification des Cricetidae (Mammalia, Rodentia) du Tertiaire de l'Europe. Scripta Geologica 2, 1-37.
Mein, P., Michaux, J., 1979. Une faune de petits mammiferes d'age turolien moyen

(Miocene superieur) a Cucuron (Vaucluse); donnees nouvelles sur le genre Stephanomys (Rodentia) et consequences stratigraphiques. Geobios 12, 481-485.

Mein, P., Martín-Suárez, E., Agustí, J., 1993. Progonomys Schaub, 1938 and Huerzelerimys gen. nov.(Rodentia); their evolution in Western Europe. Scripta Geologica 103, 41-64.

Minwer-Barakat, R., 2005. Roedores e insectívoros del Turoliense superior y el Plioceno del sector central de la cuenca de Guadix. Universidad de Granada, pp. 1-589.

Minwer-Barakat, R., García-Alix, A., Martin-Suarez, E., Freudenthal, M., 2005. Muridae (rodentia) from the Pliocene of Tollo de Chiclana (Granada, southeastern Spain). Journal of Vertebrate Paleontology 25, 426-441.

Minwer-Barakat, R., García-Alix, A., Agustí, J., Suarez, E., Freudenthal, M., 2009a. The Micromammal Fauna from Negratín-1 (Guadix Basin, Southern Spain): New Evidence of African-Iberian Mammal Exchanges during the Late Miocene. Journal of Paleontology 83, 854.

Minwer-Barakat, R., García-Alix, A., Martín-Suárez, E., Freudenthal, M., 2009b. Late Turolian micromammals from Rambla de Chimeneas-3: considerations on the oldest continental faunas from the Guadix Basin (Southern Spain). Neues Jahrbuch für Geologie und Paläontologie - Abhandlungen 251, 95-108.

Misonne, X., 1969. African and Indo-Australian Muridae. Musee Royal de L'Afrique Centrale - Tervuren, Belguique Annales Serie Nº 8.

Musser, G.G., 1969. Results of the Archbold expeditions. No. 91. A new genus and species of murid rodent from Celebes, with a discussion of its relationships. American Museum Novitates 2384, 1-41.

Musser, G.G., 1981a. Results Of The Archbold Expeditions. No. 105. Notes On Systematics Of Indo-Malayan Murid Rodents, And Descriptions Of New Genera And Species From Ceylon, Sulawesi, And The Philippines. Bulletin of the American Museum of Natural History 168, 225-334.

Musser, G.G., 1981b. A new genus of arboreal rat from West Java, Indonesia. ZOOLOGISCHE VERHANDELINGEN 189, 1-35.

Musser, G.G., 1982. Results of the Archbold Expeditions .No. 107. A New Genus of Arboreal Rat from Luzon Island in the Philippines. American Museum Novitates 2730, 1-24.

Musser, G.G., 1987. The occurrence of Hadromys (Rodentia, Muridae) in early Pleistocene Siwalik strata in northern Pakistan and its bearing on biogeographic affinities between Indian and northeastern African murine faunas. American Museum Novitates, 1-36.

Musser, G.G., 1990. Sulawesi Rodents: Species Traits and ChromosomesofHaeromys

minahassaeand Echiothrixleucura(Muridae:Murinae). American Museum Novitates 2989, 1-20.
Musser, G.G., Heaney, L.R., 1992. Philippine Rodents: Definitions of Tarsomys and Limnomys plus a preliminary assessment of phylogenetic patterns among native philippine murines (Murinae, Muridae). Bulletin of the American Museum of Natural History 211, 1-144.
Musser, G.G., Durden, L.A., 2002. Sulawesi rodents: Description of a new genus and species of Murinae (Muridae, Rodentia) and its parasitic new species of sucking louse (Insecta, Anoplura) (American Museum novitates). American Museum Novitates 3368, 1-50.
Musser, G.G., Smith, A.L., Robinson, M.F., Lunde, D.P., 2005. Description of a New Genus and Species of Rodent (Murinae, Muridae, Rodentia) from the Khammouan Limestone National Biodiversity Conservation Area in Lao PDR. American Museum Novitates 3497, 1-32.
Musser, G.G., Helgen, K.M., Lunde, D.P., 2008. Systematic review of New Guinea Leptomys (Muridae, Murinae) with descriptions of two new species.
Musser, G.G., Lunde, D.P., 2009. Systematic Reviews of New Guinea Coccymys and "Melomys" Albidens (Muridae, Murinae) with Descriptions of New Taxa. Bulletin of the American Museum of Natural History 329, 1-139.
Prieto, J., Rummel, M., 2009. Small and medium-sized Cricetidae (Mammalia, Rodentia) from the Middle Miocene fissure filling Petersbuch 68 (southern Germany). Zitteliana, 89-102.
Ruiz Bustos, A., Sesé, C., Dabrio, C., Padial, J.A.., 1984. Geología y fauna de micromamíferos del nuevo yacimiento del Plioceno inferior de Gorafe-A (depresión de Guadix-Baza). Estudios Geológicos 40, 231-241.
Sanz, E., Sesé, C., Calvo, J.P., 1992. Primer hallazgo de micromamíferos de edad Turoliense en la cuenca de Madrid. Estudios Geológicos 48, 171-178.
Sesé, C., 1980. in: Aguirre, E. (Ed.). Universidad Complutense de Madrid, Servicio de Publicaciones, pp. 1-406.
Sesé, C., López-Martínez, N., 1981. Los micromamiferos Vallesiense Inferior Los Valles de Fuentidueña Segovia. Estudios Geológicos 37, 369-381.
Sesé, C., 2003. Paleontologia y bioestratigrafia del mioceno continental de la Cuenca de Calatayud (Zaragoza): nuevos yacimientos de micromamiferos. Estudios Geológicos 59, 249-264.
Sesé, C., 2004. Nuevos datos de micromamíferos del Mioceno Medio en la Cuenca de Montalbán: el yacimiento de Bañón (Provincia de Teruel, España), Miscelánea en homenaje a Emiliano Aguire. Paleontología. Museo Arqueológico Regional.
Tate, G.H.H., 1936. Some Muridae of the Indo-Australian Region. Contained in

Bulletin of the American Museum of Natural History, Volume 72, Issue 6 pages 501-728. Bulletin of the American Museum of Natural History 72, 501-728.

van de Weerd, A., 1976. Rodents fauna of the Mio-Pliocene continental sediments of the Teruel-Alfambra region, Spain. Utrecht Micropaleontological Bulletins. Special publication. Utrecht 2, 185.

Wang, Y., Jin, C.Z., Zhang, Y.Q., Qin, D.G., 2010. Murid rodents from the Homo sapiens Cave of Mulan Mountain, Chongzuo, Guangxi, South China. Acta Anthropologica Sinica 29, 303-316.

Wessels, W., 2009. Miocene rodent evolution and migration Muroidea from Pakistan, Turkey and Northern Africa, Faculty of Geosciences. Utrecht University, Utrecht (The Netherlands).

Wessels, W., Reumer, B.M., 2009. Democricetodon and Megacricetodon from Sandelzhausen. Paläontologische Zeitschrift 83, 187-205.
